# Transcriptional Heterogeneity and Differential Response of Rod Photoreceptor Pathway Uncovered by Single‐Cell RNA Sequencing of the Aging Mouse Retina

**DOI:** 10.1111/acel.70001

**Published:** 2025-02-15

**Authors:** Laura Campello, Matthew J. Brooks, Benjamin R. Fadl, Hyo Sub Choi, Soumitra Pal, Anand Swaroop

**Affiliations:** ^1^ Neurobiology, Neurodegeneration and Repair Laboratory, National Eye Institute National Institutes of Health Bethesda Maryland USA

**Keywords:** aging, bipolar cells, molecular heterogeneity, photoreceptor, retina, single cell RNA‐seq, transcription, vision impairment

## Abstract

Visual function deteriorates throughout the natural course of aging. Age‐related structural and functional adaptations are observed in the retina, the light‐sensitive neuronal tissue of the eye where visual perception begins. Molecular mechanisms underlying retinal aging are still poorly understood, highlighting the need to identify biomarkers for better prognosis and alleviation of aging‐associated vision impairment. Here, we investigate dynamics of transcriptional dysregulation in the retina and identify affected pathways within distinct retinal cell types. Using an optimized protocol for single‐cell RNA sequencing of mouse retinas at 3, 12, 18, and 24 months, we detect a progressive increase in the number of differentially expressed genes across all retinal cell types. The extent and direction of expression changes varies, with photoreceptor, bipolar, and Müller cells showing the maximum number of differentially expressed genes at all age groups. Furthermore, our analyses uncover transcriptionally distinct, heterogeneous subpopulations of rod photoreceptors and bipolar cells, distributed across distinct areas of the retina. Our findings provide a plausible molecular explanation for enhanced susceptibility of rod cells to aging and correlate with the observed loss of scotopic sensitivity in elderly individuals.

## Graphical abstract

Healthy aging is accompanied by transcriptional dysregulation in retinal cells, leading to alterations in key molecular pathways and cellular responses, with the scotopic pathway being particularly affected. We show that rod photoreceptors, essential for initiating scotopic (low‐light) vision, exhibit selective vulnerability to aging‐associated neurodegeneration and comprise two transcriptionally distinct subpopulations: (1) phototransduction‐efficient rods (PER), defined by enhanced light detection functions and mitochondrial ATP synthesis and (2) synaptic transmission‐efficient rods (SER), enriched in neurotransmission processes.

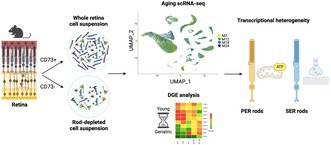

## 1 Introduction

Humanity has been intrigued by the aging process since ancient times. None of the theories put forth to explain aging have fully grasped the wide‐spread yet highly variable transformations with advanced age (Johnson, Shokhirev, and Shoshitaishvili 2019). Aging is a universal biological phenomenon that affects all organisms, resulting in gradual decline of physiological functions and enhanced vulnerability to diseases. Progressive deterioration in capabilities to do distinct tasks transpires throughout the lifespan of an organism and is relatively conserved across species (Singh et al. 2019; Lopez‐Otin et al. 2023). Importantly, age‐related changes involve intricate tissue‐ and cell type‐specific alterations in biological processes that are categorized as hallmarks of aging. At least 12 hallmarks are recognized for mammalian aging; these include genomic instability, telomere attrition, epigenetic alterations, loss of proteostasis, disabled macroautophagy, deregulated nutrient‐sensing, mitochondrial dysfunction, cellular senescence, stem cell exhaustion, altered intercellular communication, chronic inflammation, and dysbiosis (Lopez‐Otin et al. 2023). Given that the increase in human lifespan is outpacing healthy aging, more targeted research is needed to resolve the mystery of tissue‐specific aging in order to identify interventions for reducing undesired clinical outcomes (Partridge, Deelen, and Slagboom 2018). For instance, though aging appears to be inexorable, the pace of aging may be modified by lifestyle, environmental or genetic factors as well as by therapeutic interventions (Mihaylova et al. 2023; Singh et al. 2023; Yang et al. 2023; Browder et al. 2022).

A progressive decline of sensory functions in aging individuals reflects a broader decrease in neuronal activity (Salat 2011). The predominant sensory system in humans is vision. Advanced age has a profound impact on the retina, the light‐sensitive neuronal tissue lining the inner surface of the eye where the vision starts (Dowling 2012). Social and economic impact of vision loss is expected to rise dramatically due to an exponential growth of the aging population (Kehler 2019). Psychophysical and physiological studies in humans have described lower visual acuity, impaired dark adaptation, and reduction in contrast sensitivity (especially under low luminance levels), as well as altered sensitivity to motion and color perception in the elderly (Owsley 2011). Time‐dependent vision deterioration can significantly diminish individual's quality of life by adversely impacting daily routines, social connections, and overall independence. In addition, structural alterations in the neural retina are closely linked to the aging process (reviewed in Campello et al. (2021)). Cellular and molecular mechanisms underlying retinal aging are not yet fully understood; however, the signatures of aging in the retina encompass a diverse set of biological processes including transcription, mitochondrial function and proteostasis (Campello et al. 2021; Weinberg et al. 2022; Mondal et al. 2024). An understanding of signature genes and pathways in distinct retinal cell types would permit better comprehension of the complexities of aging and development of interventions to slow down the progression of age‐associated vision impairment.

Compromised transcriptional integrity is a prominent hallmark of mammalian aging (Stegeman and Weake 2017; Benayoun et al. 2019; Park et al. 2009). The first report on aging human retina using slide microarrays showed changes in the expression of 24 genes related to energy metabolism, stress response, cell growth, and neuronal signaling (Yoshida et al. 2002). A decade later, a larger microarray study demonstrated the influence of age and anatomic location (macula versus peripheral retina) on the human retinal transcriptome (Cai et al. 2012). Notably, the mammalian retina is composed of six major neuronal cell types, each having a unique role in capture or processing of visual information. Therefore, whole‐retina transcriptome profiles fail to capture cell type‐specific gene expression changes that occur throughout the human lifespan. In this context, transcriptome profiling of purified mouse rod photoreceptors over time had identified expression changes in genes involved in angiogenesis, lipid/retinoid metabolism, oxidative phosphorylation, neuronal signaling, stress, and immune response (Corso‐Diaz et al. 2020; Parapuram et al. 2010).

Advances in single‐cell omics technologies have unraveled unprecedent details of heterogeneity in the aging process (He et al. 2020). The aging transcriptomes of single cells from multiple tissues, including brain, lung, muscle, and pancreas, among others, have been reported (Uyar et al. 2020), yet longitudinal in vivo studies on retinal aging at single cell resolution remain scarce. Notably, a transcriptomic atlas based on 119,520 single cells from foveal and peripheral retinas of humans and macaques has indicated a specific pattern of retinal aging based on both region and cell type, with changes progressing from the fovea to the periphery (Yi et al. 2021). However, rod photoreceptors constitute over 70% of all retinal cells in mice and humans, and preparation of single‐cell suspensions can be challenging. Photoreceptor cells possess a functionally and structurally specialized primary cilium called the outer segment (Chen et al. 2021), which often breaks during the cell dissociation protocols resulting in RNA leakage and consequently issues related to ambient RNA (Fadl et al. 2020). In this study, we report identification of age‐associated transcriptional changes not only in rod photoreceptors but also in the less‐studied non‐rod retinal cells, which are obtained by an optimized retina dissociation protocol (Fadl et al. 2020) and targeted depletion of rods using the CD73 cell surface maker. Our studies uncover surprising transcriptional heterogeneity in rod photoreceptors as well as significant aging‐related alterations in cells associated with the rod pathway, providing a molecular explanation for the visual impairment in dim light conditions reported by older adults.

## 2 Results

To investigate transcriptional changes in the aging retina, we conducted a comprehensive longitudinal study, incorporating samples from mice across their entire lifespan and integrating multiple independent datasets. We generated whole retina (WR) single‐cell suspensions, as well as CD73‐negative (CD73N) suspensions, in which rods were selectively depleted using the CD73 surface marker. Since most mammalian retinas are rod‐dominated, with rods making up over 70% of all retinal cell types, our CD73N cell suspensions effectively enriched the sample with non‐rod retinal cells. Unlike humans, who possess trichromatic color vision, the mouse retina contains two types of cone opsins, S‐opsin, and M‐opsin, distributed along a dorsoventral gradient (Nadal‐Nicolas et al. 2020). To account for this distinct anatomical feature, we generated single‐cell suspensions separately from the superior and inferior regions of the mouse retina. Importantly, we performed cross‐species validation by integrating publicly available datasets from macaque and human retinas to validate our findings and examine regional differences, thereby enhancing the translational relevance of our study.

### 2.1 Single‐Cell Transcriptome of the Aging Mouse Retina

**FIGURE 1:**
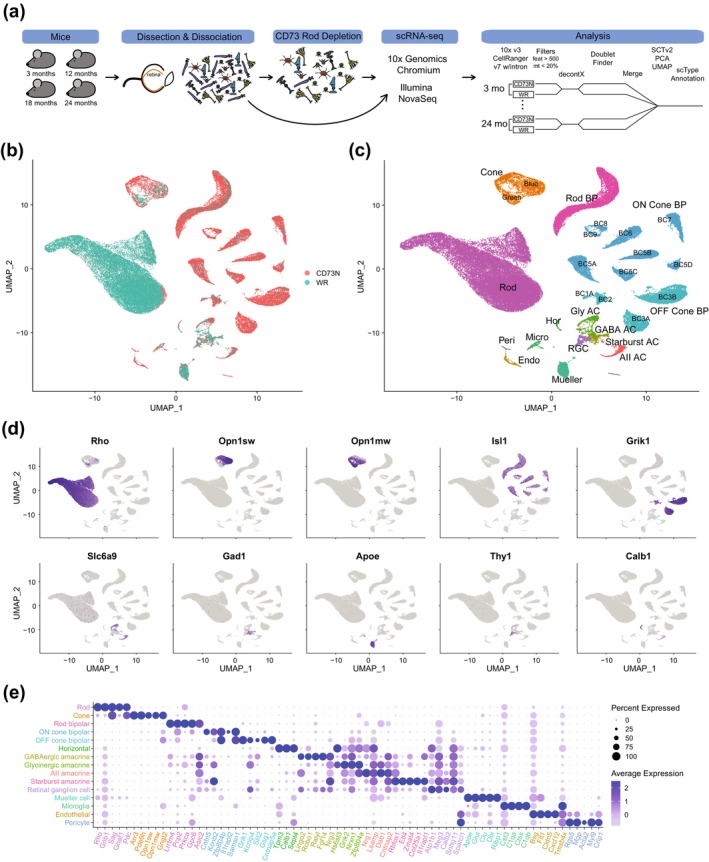
Single‐cell transcriptome profiling of aging mouse retina. (a) Workflow illustrating the preparation process of single‐cell suspensions from whole retinas (WR), or rod‐depleted retinal cell suspensions accomplished through CD73 cell surface marker‐mediated cell depletion (CD73 negative cells; CD73N) from male mice aged 3, 12, 18, and 24 months. Subsequently, the samples underwent 10x Genomics single‐cell RNA sequencing. The bioinformatic pipeline involved analyzing CellRanger data using an optimized Seurat SCT pipeline, which included the removal of ambient RNA and heterotypic doublets using DecontX and DoubletFinder, respectively. Additionally, it encompassed the annotation of retinal cell types using scType with well‐established retinal cell type markers, see methods. (b) UMAP visualization of cells obtained from WR and CD73N retinal single‐cell suspensions. (c) UMAP visualization of cell annotations. Broad cell types are depicted in larger font size, while subtypes of cones and cone bipolar cells are displayed in smaller font size. (d) Validation of clustering and cell type annotations through UMAP feature plots of cell type‐specific markers: *Rho* (rods), *Opn1sw* and *Opn1mw* (blue and green cones, respectively), *lsl1* and *Grik1* (OFF and ON bipolar cells, respectively), *Slc6a9* and *Gad1* (glycinergic and GABAergic amacrine cells, respectively), *Apoe* (Müller glia cells), *Thy1* (ganglion cells), and *Calb1* (horizontal cells). (e) Expression dot plots showing the top 5 enriched genes for each annotated cell type when compared to the entire dataset. The expression values represent log‐normalized counts. AC, amacrine cell; BC, bipolar cell subtype; BP, bipolar cell; CD73N, CD73 negative cells (non‐rod cells); Endo, endothelial cell; Feat, features (genes); GABA AC, GABAergic amacrine cell; Gly AC, glycinergic amacrine cell; Hor, horizontal cell; Micro, microglia cell; mt, mitochondria reads; Peri, pericyte; RGC, retina ganglion cell; SCTv2, sctransform version 2; UMAP, uniform manifold approximation and projection; UMI, unique molecular identifier; WR, whole retina.

After enzymatic dissociation, cell suspensions of whole retina (WR) and those after CD73‐depletion (CD73N) were processed for scRNA‐seq analysis (Figure 1a). Over 95% rod depletion allowed us to explore underrepresented cell types (Figure 1b,c). Gene expression was quantified by the total unique molecular identifier (UMI) count, which was constant among various sample preparation protocols, WR and CD73N retinal cells and time points (Figure S1). After filtering the data for high‐quality cells (Figure 1a and Figure S1), we collected single‐cell transcriptomic profiles of 71,632 cells (Figure S1) and detected a median of ~3000 genes per cell for non‐rod retinal cells (Figure S1). This number was ~1000 in whole retina cell suspensions (Figure S1). Unsupervised clustering and annotation revealed 35 distinct clusters representing the diversity of the retina, with 6 major neuronal types, glial cells, endothelial cells, and pericytes, and indicating no cell type bias in all datasets (Figure 1c and Figure S1). Depletion of CD73+ cells led to a significant enrichment of all non‐rod cell types, with rod and cone bipolar cells showing 10‐ and 15‐fold enrichment, respectively (Figure S1). We further validated our analysis by examining the expression of known cell type‐specific markers (Figure 1d,e and Table S1). Figure S1 displays the number of cells per sample, along with UMI and genes per cell, as well as UMAPs for the Sup/Inf dataset.

### 2.2 Age Influences Retinal Cell Transcriptomes

**FIGURE 2:**
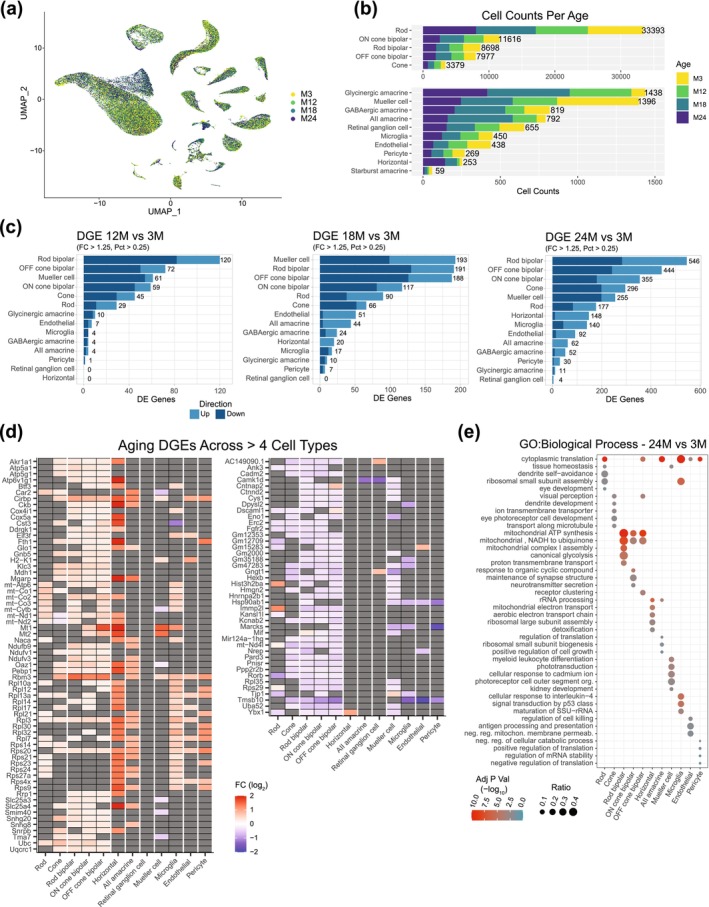
Differences in the retinal transcriptome during aging. (a) UMAP visualization of retinal cells from mice aged 3, 12, 18, and 24 months, with colors representing the age of the sample. (b) Age stratified histogram of cell counts in broadly annotated cell types. (c) Histograms depicting the number of genes with differential gene expression (DGE) (cut‐off details in Materials and Methods) in cells from 12‐, 18‐, and 24‐month‐old mice compared to 3‐month‐old mice, stratified by the direction (up or down) of expression change. (d) Genes upregulated at 24 months compared to 3 months across more than 4 broad cell types (left). Downregulated genes are shown in the right panel. Gray boxes indicate no significant change. (e) Functional gene enrichment results for Gene Ontology (GO) biological process ontology terms in the comparison between 24‐month‐old and 3‐month‐old samples. DGE, differential gene expression; FC, fold‐change; GO, gene ontology; Pct, percentage present.

We next examined the variation in cell composition across different ages. For a majority of cell types, we did not observe any significant change in the overall abundance and/or relative proportions of retinal cells over time (Figure 2a,b). Then, we assessed age‐associated changes in gene expression within distinct cell populations. DGE analysis revealed an increase in the number of differentially expressed genes (DEGs) with age (Figure 2c and Table S2). This trend appears to be a common feature across all retinal cells; however, further validation is needed due to limitations in capturing small cell populations, which resulted in lower coverage of certain cell types, such as horizontal cells, retinal ganglion cells, and some subtypes of amacrine cells. The magnitude and direction of the changes were dependent on individual cell type and age studied. Overall, DEGs from middle‐aged mouse retina (12‐month) exhibited a broader downregulation, whereas DEGs from retinas of older mice (18‐ and 24‐month) displayed a switch in pattern, as evidenced by several genes showing high expression (Figure 2c). Photoreceptors, bipolar and Müller glia cells had the highest number of DEGs in all ages studied (Figure 2c). Notably, many observed changes were shared across different cell types (see Figure 2d, showing a subset of aging‐associated genes). Next, we investigated changes in aging‐related cellular pathways by performing Gene Ontology (GO) term enrichment analysis (Gene Ontology Consortium et al. 2023) of the DGEs associated with each cell type (Figure 2e, Figure S2, and Table S3). We identified many shared as well as cell‐type specific aging‐related pathways. In total, 540 reduced‐redundancy pathways (382 unique) were significantly enriched (adj *p* < 0.05) at all the timepoints investigated; of these, 119 were significantly enriched in at least 2 cell types at the timepoint interrogated, whereas the remaining were unique for specific cell populations. Of the 119 pathways altered in multiple cell types, 77 exhibited the same directionality regardless of the cell type (38 were upregulated and 39 downregulated), whereas the direction of change in the remaining 42 varied across cell types. The top 5 enriched biological processes over time are energy production, synaptic components, neurotransmission, visual perception, and RNA and translation processes (Figure 2e and Figure S2).

Notably, we identified a significant number of genes encoding ribosomal subunits (*Rpl* and *Rps* genes) that were differentially expressed during aging across cell types (Figure 2d). This observation suggests heterogeneity in ribosomal stoichiometry and composition, with age‐related changes potentially serving as compensatory mechanisms. These adaptations may facilitate the translation of anti‐aging mRNAs, such as those involved in mitochondrial function, cellular metabolism, DNA repair, and RNA‐binding proteins, thereby enhancing cellular resilience and promoting longevity. Alternatively, the dysregulation of these genes during aging could impair ribosome assembly, resulting in the production of orphan proteins prone to aggregation, which may be detrimental to retinal cells. Further studies are essential to elucidate the role of ribosomal stoichiometry and composition heterogeneity and their contributions to aging. Ribosome heterogeneity is an emerging area of interest within the scientific community; however, its significance and implications remain poorly understood, especially in the contexts of aging and disease (reviewed in Islam and Rallis (2023)).

Considering vision impairment under low light conditions at an advanced age and recognizing that the identified transcriptional changes may contribute to retinal aging phenotype, we focused on detailed analysis of rod photoreceptors and rod bipolar cells—two of the primary neurons associated with the dim light vision.

### 2.3 Transcriptional Heterogeneity Within the Rod Population

**FIGURE 3:**
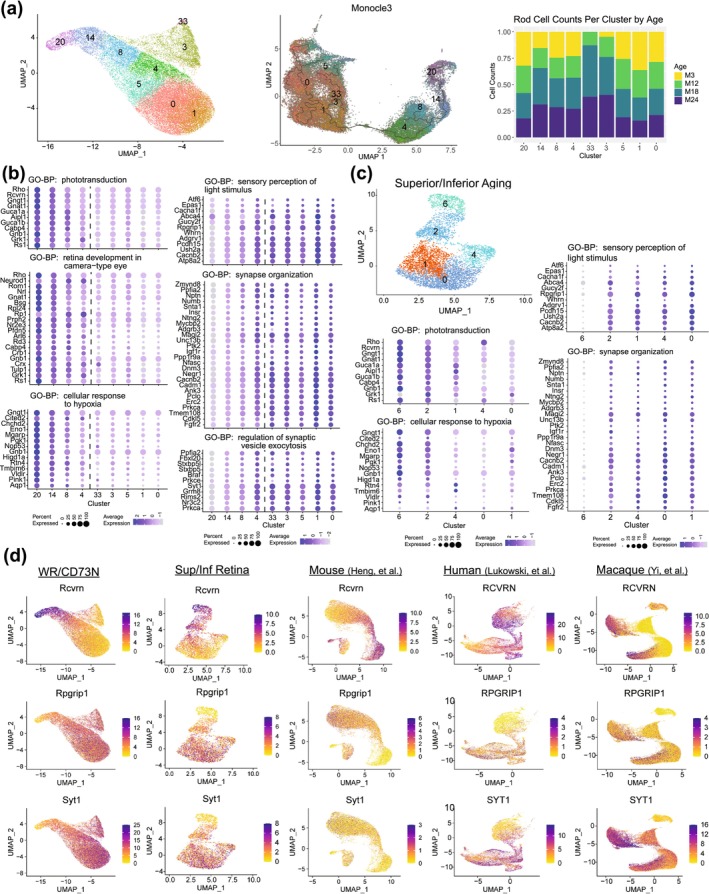
Transcriptional heterogeneity in rod photoreceptors. (a) UMAP projection of the rod photoreceptor cell sub‐clusters in WR/CD73N dataset determined by the Louvain algorithm in Seurat (left) and alternative clustering and UMAP using Monocle3 of the rod clusters identified in Seurat (middle). Cell population in each rod cluster for each age (right). (b) Dot plots highlighting differential expression of genes identified in PER versus SER comparison and enriched in selected Gene Ontology terms (biological process). Left column shows terms enriched in PER and right column shows terms enriched in SER. Average expression values are scaled. A vertical dashed line separates PER clusters (4, 8, 14, 20) from SER clusters (0, 1, 5, 3, 33). (c) UMAP projection of rod clusters identified in the second independent scRNA aging retina dataset obtained from superior and inferior (Sup/Inf) retinal punches. Expression dot plots of the enriched ontology terms identified as significant in (b) are populated with data from superior and inferior retina aging data to highlight the presence of rod heterogeneity in the Sup/Inf dataset. Average expression values are scaled. (d) UMAP projections showing the presence of marker genes for PER (*Rcvrn*) and SER (*Rpgrip1* and *Syt1*) rod photoreceptors in our two current retina aging datasets (WR/CD73N and Sup/Inf), as well as publicly available datasets including adult mouse (Heng et al. 2019), human (Lukowski et al. 2019), and macaque (Yi et al. 2021) retinas. Expression values (color) represent normalized counts. CD73N, CD73 negative cells (non‐rod cells); GO‐BP, gene ontology—biological process; PER, phototransduction‐efficient rods; SER, synaptic transmission‐efficient rods; Sup/Inf, superior/inferior retina; WR, whole retina.

Further investigations of the rod transcriptome at a higher resolution identified 9 subclusters, revealing the existence of transcriptionally distinct, heterogeneous subpopulations of rod photoreceptors (Figure 3a, left). The subclusters were then subdivided into two populations based on the pseudo‐time ordering of cells (Figure 3a, middle), composed of clusters 20, 14, 8 and 4, and 33, 5, 3, 1 and 0, respectively (Figure 3a). In this instance, pseudo‐time was not associated with age but instead reflected divergent gene expression between the two rod populations relating to several key biological processes. DGE (Table S4) and functional gene enrichment (Table S5) analyses of the two populations revealed that variations among the clusters stemmed from the significance of the predominant biological processes within each group. Phototransduction‐efficient rods (PER) (including clusters 20, 14, 8 and 4) exhibited high expression of genes associated with light detection, cellular response to hypoxia, and mitochondrial ATP synthesis (Figure 3b left column, Figure S3 and Table S5), whereas synaptic transmission‐efficient rods (SER) (consisting of clusters 33, 5, 3, 1, and 0) displayed high expression of genes associated with neurotransmission (Figure 3b right column, Figure S3 and Table S5). Another remarkable observation was that clusters 3 and 33 were unequivocally age‐related clusters, given the absence or little representation of rod cell counts in 3‐ and 12‐month‐old retinas (Figure 3a, right). Transcriptional heterogeneity of rods was also evident in WR samples from Sup/Inf aging dataset (Figure 3c and Figure S4). To ensure the validity of the rod heterogeneity observed in this study, we cross‐referenced marker genes, identified through stringent filtering of DGE results from the PER cluster 20 and SER cluster 1 (Table S6), with those from publicly available single‐cell and single‐nuclear transcriptomic studies. Notably, the dynamics of the rod transcriptome was also evident in published retinal datasets from mouse, macaque, and human (Heng et al. 2019; Lukowski et al. 2019; Yi et al. 2021; Fadl et al. 2020; Peng et al. 2019; Yan et al. 2020) (Figure 3d and Figure S3). We then investigated the potential regional distribution of PER and SER rods in the retina by examining a panel of gene markers (Table S6) in datasets containing rod cells from different retinal regions. This analysis included rods from our mouse superior and inferior retina dataset (Figure S5), and publicly available datasets for the human fovea, macula, and peripheral retina (Liang et al. 2023) (Figure S5) and the macaque fovea and peripheral retina (Yi et al. 2021; Peng et al. 2019) (Figure S6). We did not observe notable differences in the expression of PER or SER marker genes between the superior and inferior regions of the mouse retina. However, there appears to be a slight trend toward higher expression of PER markers in the central regions of the human retina (fovea and macula) compared to the peripheral areas. A similar pattern is also observed in the macaque retina, although to a lesser extent.

### 2.4 Aging Differences in Rod Populations

**FIGURE 4:**
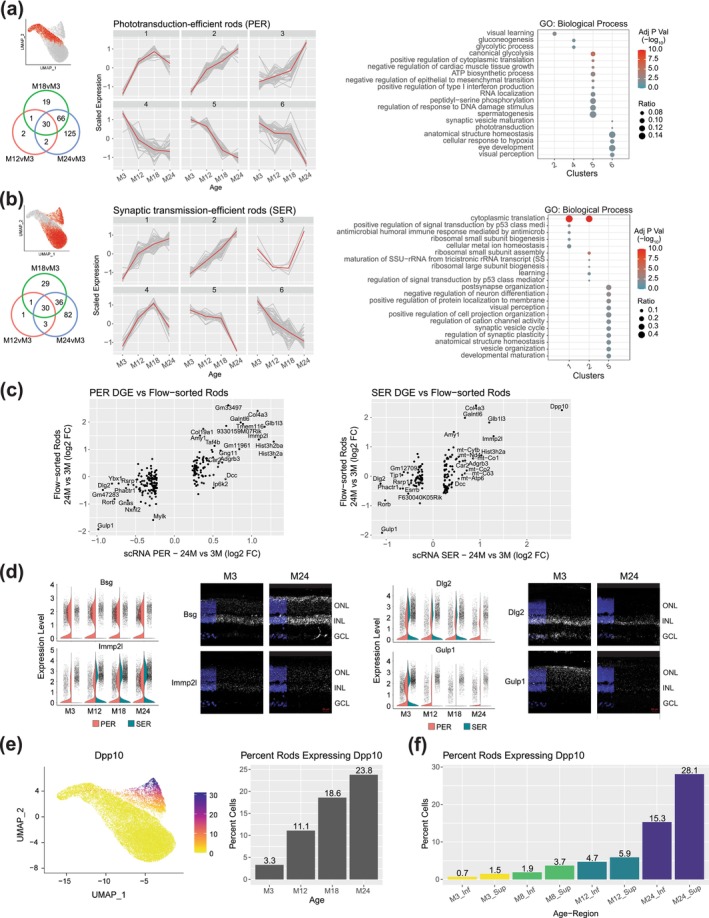
Molecular signatures in aging rod photoreceptors. (a) DGE results in PER. UMAP projection highlighting PER cell population in rods (top left). Venn diagram displaying the number of significant DGEs in PER cells for each age comparison: 12, 18, and 24 months versus 3 months (bottom left). Progression of scaled gene expression over age of 245 DGE genes clustered for similar patterns. The red line indicates the median expression of the cluster (middle). Enrichment analysis of GO biological process enrichment for the DGE gene clusters (right). (b) DGE results in SER. UMAP projection highlighting SER cell population in rods (top left). Venn diagram displaying the number of significant DGEs in SER cells for each age comparison: 12, 18, and 24 months versus 3 months (bottom left). Progression of scaled gene expression over age of the 192 DGE genes clustered for similar patterns. The red line indicates the median expression of the cluster (middle). Enrichment analysis of GO biological process for the DGE gene clusters (right). (c) Comparative analysis of PER (left) and SER (right) 24‐month versus 3‐month DGE results alongside flow‐sorted rod aging samples from Corso‐Diaz et al. 2020. (d) Selected gene expression plots showing cell expression distribution from PER and SER over aging. Images show the validation of selected genes through in situ hybridization for both 3‐ and 24‐months retinas. (e) UMAP projection of *Dpp10* normalized expression counts for the entire rod population (left) and the percentage of rods expressing *Dpp10* at each age (right). (f) Percentage of rod cells expressing *Dpp10* across age groups and retinal regions in the Sup/Inf dataset. DGE, differential gene expression; FC, fold‐change; GCL, ganglion cell layer; GO, gene ontology; Inf, inferior retina; INL, inner nuclear layer; ONL, outer nuclear layer; PER, phototransduction‐efficient rods; SER, synaptic transmission‐efficient rods; Sup, superior retina.

To ensure that transcriptomic changes associated with age are not confounded by rod heterogeneity, DGE and gene enrichment analyses were conducted separately for PER and SER subsets within the WR/CD73N dataset. The PER subset included 245 significant DEGs (Figure 4a and Table S7), whereas the SER subset showed 192 DEGs (Figure 4b and Table S9) in total for the 24‐, 18‐, or 12‐month versus 3‐month comparisons. To investigate the differences in dynamic expression changes during aging of the PER and SER cells, the scaled average expression profiles at each age for the 245 significant DEGs in PER and the 192 significant DEGs in SER were hierarchically clustered and grouped into six clusters separately (Figure 4a,b) based on the observed progression patterns. In PER, most of the significantly downregulated pathways in early (3–12 months) and intermediate (12–18 months) ages are enriched for energy regulation in glycolysis and ATP synthesis, and stress with response to DNA damage (clusters 4 and 5) (Figure 4a, Figure S7, and Table S8). Interestingly, glycolytic genes were downregulated early, from 3 to 12 months, whereas genes associated with ATP production were downregulated later, from 12 to 18 months. Pathways that remained unaltered until the late stage (18–24 months, cluster 6) included phototransduction and visual perception, hypoxia, and homeostasis. In SER, upregulated pathways (clusters 1 and 2) are associated with cytoplasmic translation, ion homeostasis, and p53 signal transduction genes (Figure 4b, Figure S7, and Table S10), whereas pathways downregulated in SER implicated synapse, visual perception, and homeostasis dysregulation. Importantly, the genes associated with glycolysis and ATP production in PER cells were not significantly altered during aging in SER cells.

Our findings uncover alterations in gene expression in rod photoreceptors throughout the aging process, concordant with downregulation of the GO terms associated with visual function and synaptic transmission (Figure 4a,b). A comparison of DEGs identified in PER and SER subsets from 24‐month retinas with publicly available age‐matched bulk RNA‐seq profiles of flow‐sorted rods (Corso‐Diaz et al. 2020) confirmed the most significant changes during aging (Figure 4c). We then performed fluorescent in situ hybridization to validate expression changes for a subset of shared and cell‐type specific aging‐related DEGs. Nine target genes were selected by their level of gene expression changes between the 3‐ and 24‐month‐old retinas and their relevance to the aging process; these include *Bsg*, *Cacna2d1*, *Dlg2*, *Dpp10*, *Drd4*, *Gulp1*, *Immp2l*, *Rbm3*, and *Tafa3*. We validated transcriptional changes in the aging retina for *Bsg*, *Dlg2*, *Gulp1*, and *Immp2l* by in situ hybridization (Figure 4d). The *Dpp10* gene is particularly intriguing, with almost no expression detected in young rods but displaying high expression in aged SER rods (Figure 4e and Figure S7). Furthermore, *Dpp10* presents a notably distinct spatial expression pattern, with higher expression in the superior compared to the inferior retina, consistently observed across different age groups (Figure 4f).

### 2.5 Gene Expression Differences in Aging Bipolar Cells

**FIGURE 5:**
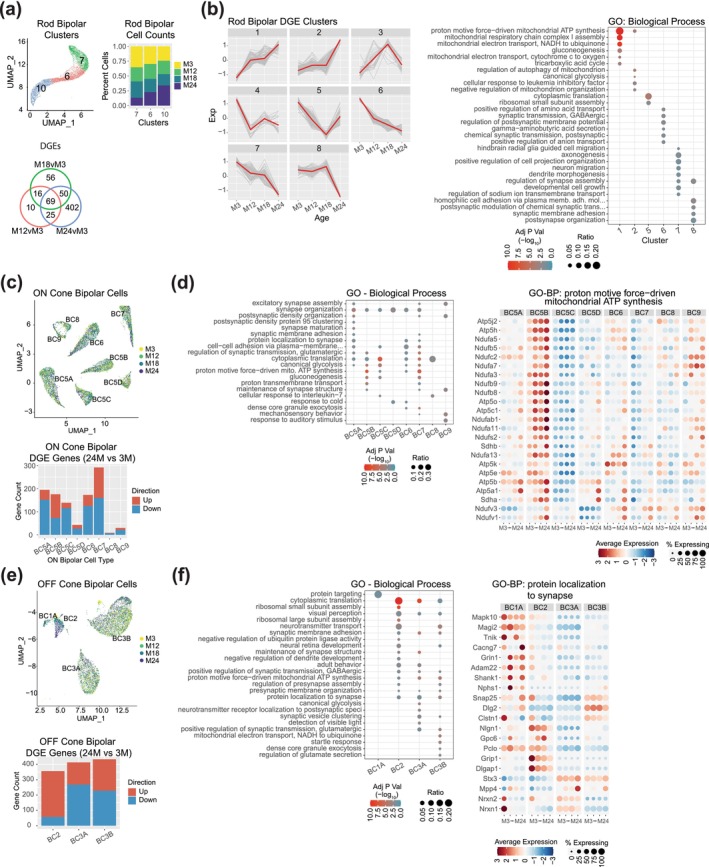
Gene expression differences in aging bipolar cells. (a) UMAP projection highlighting rod bipolar cell clusters (top left) and percentage of cells per cluster at each age (top right). Venn diagram displaying the number of significant DGEs for each age comparison: 12, 18, and 24 months versus 3 months (bottom). (b) Progression of scaled gene expression over age of the 628 DEG genes clustered for similar patterns. The red line indicates the median expression of the cluster (left). Enrichment analysis of GO biological process enrichment for the DGE gene clusters (right). (c) UMAP visualization of ON cone bipolar cells colored by age of sample and labeled by sub‐cell type (top). Number of significant DGEs in each sub‐cell type in the 24‐month versus 3‐month comparison (bottom). (d) GO biological process enrichment of the 24‐month versus 3‐month DGEs for each sub‐cell type (left). Expression dot plot of the DGE genes for the selected GO term shown at each age in each sub‐cell type. Scaled average expression is shown (right). (e) UMAP visualization of OFF cone bipolar cells colored by age of sample and labeled by sub‐cell type (top). Number of significant DGEs in each sub‐cell type in the 24‐month versus 3‐month comparison (bottom). (f) GO biological process enrichment of the 24‐month versus 3‐month DGEs for each sub‐cell type (left). Expression plot of the DGE genes for the selected GO term shown at each age in each sub‐cell type. Scaled average expression is shown (right). DGE, differential gene expression; GO, gene ontology; GO‐BP, gene ontology—biological process.

Transcriptomic changes in rod and cone bipolar cells were assessed using previously reported markers (Shekhar et al. 2016), and the cone sub‐cell types were named accordingly. Rod bipolar cells consisted of 3 UMAP clusters (Figure 5a, top left), showing a differential gradient of age composition (Figure 5a, top right), with cluster 7 enriched with 3‐month cells, cluster 6 having a similar composition of cells across ages, and cluster 10 enriched with 24‐month cells. A total of 628 DEGs (Table S2) identified from rod bipolar cells by comparing the 24‐, 18‐, or 12‐month versus 3‐month retina (Figure 5a, bottom) could be grouped into 8 expression pattern clusters (Figure 5b, left; Table S11) that were then used in GO functional gene enrichment (Figure 5b, right; Figure S8 and Table S12). The pathways showing an early increase in expression (cluster 1) comprised of mitochondrial complex I and IV and gluconeogenesis, whereas the late‐increasing pathways (cluster 2) included glycolysis, autophagy, and organization of the mitochondria. The pathways showing early downregulation (clusters 5 and 6) consisted of cytoplasmic translation and synaptic transmission, whereas the late changers (clusters 7 and 8) contained axonogenesis and synaptic assembly, adhesion, and organization.

DGE analysis of ON cone bipolar cells was then performed individually for each of the identified sub‐cell type (BC5A, BC5B, BC5C, BC5D, BC6, BC7, BC8, and BC9) (Figure 5c upper, Table S13). Overall, the number of cells in each sub‐cell type and for each age was consistent and revealed little sampling bias associated with age (Figure S8). Cell type BC7 revealed the most (292) aging‐related DEGs in 24‐ versus 3‐month comparison (Figure 5c lower, Table S13), followed by BC5A, B, C, and BC6 with 150–200 DEGs each, and the lower sampled (Figure S1) cells—BC5D, BC8, and BC9—with < 50 DGEs each. The enriched GO biological process pathways for each cell type (Figure 5d left, Table S14) uncovered multiple synapse‐related categories: ATP synthesis, cytoplasmic translation, glycolysis, and gluconeogenesis, and cell–cell adhesion (Figure 5d right, Figure S8).

OFF cone bipolar cell aging analysis was performed individually for each of the identified sub‐cell type (BC1A, BC2, BC3A, and BC3B) (Figure 5e upper). BC1A and BC2 showed significant timepoint bias in our data (Figure S8) with 74.6% of BC1A cells originating from the M18 timepoint. BC2 cell numbers increased significantly at the M18 and M24 timepoints, each consisting of ~40% of BC2 cells with M3 and M12 making up 8.4% and 11.9% of the BC2 population, respectively. The most aging‐related DEGs were evident in the 24‐ versus 3‐month comparison with BC3A and BC3B having 413 and 433 significant genes, respectively, (Figure 5e lower, Table S13). Cell type BC1A had no significant DEG at 24‐ versus 3‐month comparison, yet the 18‐ versus 3‐month comparison revealed 19 significant DEGs. Similar to the ON cone bipolar cells, GO biological process analyses for each cell type (Figure 5f left, Table S14) demonstrated multiple synapse‐related categories, ATP synthesis, cytoplasmic translation, glycolysis, and neurotransmitter transport (Figure 5f right, Figure S8).

## 3 Discussion

Our study was designed to gain insights into the current knowledge gap concerning molecular mechanisms and cellular pathways associated with retinal aging. It has long been recognized that rod photoreceptors are particularly vulnerable to the effects of aging, resulting in a notable decline in scotopic vision (Curcio et al. 1993; Owsley 2011; Jackson, Owsley, and Curcio 2002). Importantly, functional changes in the aging retina correlate to the histopathological observations of rod loss in aging (Gao and Hollyfield 1992; Curcio et al. 1993; Curcio, Owsley, and Jackson 2000). Here, we have characterized the dynamics of age‐related gene expression changes in retinal cells at single‐cell resolution and uncovered significant transcriptional dysregulation in rod photoreceptors and bipolar cells. Our data are therefore consistent with alterations in rod function observed in elderly individuals through psychophysical tests.

### 3.1 Rod Photoreceptor Heterogeneity

scRNA‐seq has enabled us to reveal the existence of heterogeneous subpopulations within established retinal cell types. Yet, previous studies have not exposed the transcriptional heterogeneity within rod photoreceptors despite some previous suggestions. Early studies on rat retinal development indicated the existence of two intrinsically distinct populations of rod precursors that constitute the two phases of rod photoreceptor differentiation (Morrow, Belliveau, and Cepko 1998). A population of rhodopsin‐positive and recoverin‐negative rod photoreceptors has also been reported in primary cultures of postnatal retinal neurons (Cao et al. 2000). Furthermore, a subpopulation of rod photoreceptors in the mouse retina seems to establish direct synaptic connections with OFF cone bipolar cells, suggesting the existence of an alternative pathway alongside the conventional rod pathways (Tsukamoto et al. 2001). Detection of genetic and epigenetic footprints of S‐cones in rod photoreceptors of mouse but not zebrafish retina and cell lineage tracing also point to two independent stages of rod evolution in vertebrates (Kim et al. 2016). The presence of two subpopulations of rod photoreceptors in the human post‐mortem retina can be distinguished by expression of a long non‐coding RNA, MALAT1 expression (Lukowski et al. 2019). Another study has also reported two subtypes of rods in the human retina based on MYO9A expression and indicated that MYO9A negative rods are more susceptible to the effects of aging (Yi et al. 2021).

Our data provide strong evidence of transcriptomic heterogeneity in rod cells (Figure 3a). Re‐clustering analyses of the rod cells from several previous single cell studies (Figure 3d) clearly indicates two groups of rods. Two possible explanations exist for this transcriptomic division. Firstly, the rod cells may belong to either of two cell states in which a specific group of genes is overexpressed, and vice versa. Alternatively, the two groups may correspond to two subtypes of rods. Transcriptomic data alone cannot distinguish between these possibilities. Multimodal data, such as epigenomics, evolutionary developmental roots (evo‐devo) of transcriptome across species, and especially for rod cells, morphology, shape, and inter‐cell connectivity may help resolve this dichotomy. Nonetheless, we favor the second hypothesis of two distinct rod populations in mouse retina as elaborated previously. Considering that cones have higher energy demand than rods (Okawa et al. 2008), phototransduction efficient rods (PER) with high expression of genes involved in light detection and mitochondrial ATP synthesis could evolutionarily be derived from S cones. Further investigations would be necessary to test this hypothesis. The second subpopulation of rods, we called synaptic transmission efficient rods (SER), may represent alternative signaling pathways for visual transduction in the mammalian retina (Tsukamoto et al. 2001; Hack, Peichl, and Brandstatter 1999; Li, Chen, and DeVries 2010).

The advent of the adaptive optics imaging has enabled researchers to image the photoreceptor mosaic in the living retina, allowing the investigation of variations in the reflectivity of individual cone and rod photoreceptors (Jonnal et al. 2007; Cooper et al. 2011). It is possible that transcriptomic distinctions may be indicative of dynamic fluctuations in physiological activity within photoreceptors, potentially originating from timely variances in phototransduction and/or neurotransmission.

### 3.2 Candidate Biomarkers of Retinal Aging

In this study, we have also discovered a useful set of genetic markers associated with aging of distinct retinal cells and observed agreements between the scRNA‐seq data and in situ hybridization results for *Immp2l, Gulp1*, *Dlg2*, and *Bsg*. Our results demonstrate overexpression of *Immp2l*, which encodes the inner mitochondrial membrane peptidase subunit 2, in the aging rods as well as a reduction in the bipolar cells over time. *Immp2l* mouse mutants exhibit multiple age‐associated phenotypes, thought to be driven by increased generation of mitochondrial reactive oxygen species (ROS) (George et al. 2011). In addition, suppressing *Immp2l* expression in cell cultures leads to cellular senescence (Yuan et al. 2018). *Immp2l* regulates the activity of apoptosis inducing factor (AIF) by cleaving it to its pro‐apoptotic form under oxidative stress (Yuan et al. 2018). In the aging rods, higher expression of *Immp2l* may indicate removal of defective rods via apoptosis; however, since multiple photoreceptors synapse with a bipolar cell, downregulation of *Immp2l* may reflect sparing of rod bipolar cells under age‐associated stress conditions to maintain signaling from functional and relatively healthy rods.

Our data show a downregulation of *Gulp1* and *Dlg2* in rods of the 24‐month‐old retina. Conversely, the *Bsg* gene expression is higher in the aged retinas. *Gulp1* encodes an engulfment adaptor protein that is involved in the phagocytosis of apoptotic cells (Hayashi et al. 2020). A deficiency in GULP1 protein reportedly increases nuclear factor E2–related factor 2 (NRF2) activity to resist cisplatin‐induced oxidative stress (Hayashi et al. 2020). GULP1 also interacts with amyloid precursor protein to produce amyloid beta peptide (Hao et al. 2011). In the context of the aging retina, downregulation of *Gulp1* expression may decrease breakdown of apoptotic cells or act as a compensatory mechanism to induce stress response pathways. *Dlg2* encodes the postsynaptic scaffolding protein that interacts with multiple receptors and channels (Griesius et al. 2022). Expression of *Dlg2* is downregulated in inflammatory gastrointestinal conditions, such as ulcerative colitis (Keane et al. 2022), suggesting a regulatory role of *Dlg2* in inflammatory response. Considering that para‐inflammation and inflammation exist in the aging retina and age‐associated retinal conditions, such as age‐related macular degeneration (Xu, Chen, and Forrester 2009; Campello et al. 2021), it is plausible that the downregulation of *Dlg2* expression influences inflammatory state of the aging retina. *Bsg* encodes basigin, a glycosylated transmembrane protein involved in multiple biological processes, including inflammation, development, and nutrient transport (Iacono et al. 2007). Loss of *Bsg* in mice leads to a decrease in rod and cone functions and early‐onset photoreceptor degeneration by week 41 of age (Hori et al. 2000). Though the role of basigin in the aging retina is unclear, basigin can likely be upregulated in the aging retina as a compensatory response to age‐related homeostatic imbalance.

A remarkable finding in our study is the presence of an aging‐associated rod photoreceptor subpopulation that expresses *Dpp10* prominently at 12, 18, and 24‐months age, with almost no expression in photoreceptors at 3‐months (Figure 4e and Figure S7). Although we were unable to immunolocalize DPP10 protein in the aging retina due to the lack of suitable antibodies, our observation is supported by *Dpp10* expression in published aging flow‐sorted rod photoreceptor data (Corso‐Diaz et al. 2020) that is integrated into our analysis (see Figure 4c, right plot). DPP10 (dipeptidyl peptidase like 10) is a transmembrane glycoprotein involved in assembling voltage‐activated potassium channels (Kv channels) and modulating neuronal excitability in the brain (Wang, Cheng, and Tsaur 2015; Jerng, Qian, and Pfaffinger 2004; Brueggemann, Gentile, and Byron 2013). Abnormal expression of DPP10 in the neurofibrillary tangles and plaque‐associated dystrophic neurites of the human brain has been associated with neurodegenerative disorders such as Alzheimer's Disease and other major tauopathies (Chen, Gai, and Abbott 2014). Kv channels are intensively regulated by metabotropic receptors (Fernandez‐Fernandez and Lamas 2021) and glutamate transporters (Caminos, Vaquero, and Martinez‐Galan 2015). In the retina, Kv7.5/ VGluT1 interactions exist and change with aging and degeneration (Caminos, Vaquero, and Martinez‐Galan 2015). Considering the presence of *Dpp10* expression within the synaptic transmission‐efficient rods (SER) sub‐cluster, it is plausible that DPP10 modulates the neuronal excitability‐mediated potassium currents, thereby adjusting the signaling transmission from rods to rod bipolar cells.

### 3.3 Transcriptional Noise and Cellular Senescence in the Aging Retina

Aging is a complex process often associated with an increase in transcriptional noise. However, whether this phenomenon is an inherent characteristic of the aging process remains uncertain. Until recently, studies suggested that transcriptional noise could arise from extrinsic sources, such as cell–cell or cell‐matrix interactions, as well as chemokines diffusing in the extracellular environment. Alternatively, intrinsic noise may stem from the inherent variability of intracellular and intranuclear fluctuations in molecules or from alterations in the chromatin environment, including accessibility and the limited availability of transcription factors (Elowitz et al. 2002; Swain, Elowitz, and Siggia 2002; Kaern et al. 2005). Expression variability can also result from transcriptional bursting, that is, the stochastic activation and inactivation of transcription (Suter et al. 2011; Fukaya, Lim, and Levine 2016; Fritzsch et al. 2018). However, recent studies point to a different scenario, providing evidence of non‐random transcriptional changes in aging (Ibañez‐Solé et al. 2022; Bartz et al. 2023) and demonstrating the existence of gene‐to‐gene coordinated changes in transcriptional expression (Levy et al. 2020). To assess transcriptome variability across cells for each retinal cell type, we calculated the mean and standard deviation of expression levels for all cells within each type and visualized the results using scatter plots (Figure S9). We did not observe any significant increase in cell‐to‐cell variability with age. Although heteroscedasticity (variance dependent on the mean) was evident in the raw counts, Seurat normalization, which incorporates variance stabilization methods to account for confounding technical factors such as sequencing depth, effectively corrects for it, giving us confidence in applying differential expression methods to our analysis.

Another key aspect of aging is cellular senescence, a cellular response to harmful stimuli characterized by cell‐cycle arrest and the release of an inflammatory secretome (Huang et al. 2022). Notably, cellular senescence is not limited to dividing cells but is also observed in postmitotic cells. In both human neurodegenerative diseases and animal models, neurons in the central nervous system (CNS) exhibit signs of senescence (Baker and Petersen 2018; Jurk et al. 2012). In the human eye, the expression of canonical senescence markers such as p16, p21, and p53 has been documented in the retinal pigment epithelium (RPE) during aging (Chaum, Winborn, and Bhattacharya 2015) and in age‐related macular degeneration (AMD) (Lee et al. 2021; Sreekumar, Hinton, and Kannan 2020). Similarly, in the old human retina, p16 expression is upregulated in rod photoreceptors, as well as in horizontal, amacrine and ganglion cells (Lopez‐Luppo et al. 2017). Importantly, it is worth noting that research on the characterization of senescent cells in ocular tissues during healthy aging remains limited, and there is continued discussion about whether premature senescence induced experimentally in animal models could contribute substantially to the development of age‐related diseases. Moreover, the study of senescence markers should be conducted carefully, as it can lead to false‐positive interpretations. Previous studies have reported positive p16 immunoreactivity in the nucleus, or in both the nucleus and cytoplasm (Mahajan 2016). However, the consensus is that p16 staining observed solely in the cytoplasm should be considered a false‐positive result. To explore the presence of cellular senescence in the aging mouse retina, we examined the expression of canonical senescence markers across various retinal cell types at different ages (Figure S10). Overall, senescence marker expression in retinal neurons was either absent or detected in a very low percentage of cells. For instance, we did not detect expression of the p16/INK4A/CDKN2A tumor‐suppressor gene in aging rod cells, and senescence‐associated β‐galactosidase (SA‐β‐Gal, encoded by the *Glb1* gene) was found in less than 1% of rod cells. Only cells with proliferative capacity, such as endothelial cells, pericytes, microglia, and Müller cells, showed low levels of senescence markers; however, their expression remained constant throughout the lifespan of the retina, with no age‐related increase. Taken together, these results suggest there is no prominent senescence phenomenon in the healthy aging mouse retina. Nevertheless, further longitudinal studies across different species, along with the discovery of novel, retina‐specific markers of cellular senescence, may provide additional insights.

### 3.4 Limitations of the Study

Our analysis of the aging mouse retinal transcriptome profiled over 71,000 cells, which is somewhat limited in capturing small cell populations such as subtypes of retinal ganglion cells (RGCs), thereby missing information on RGC aging. An increase in the number of cells analyzed and deeper sequencing should alleviate this issue. In this context, the use of new scRNA‐seq methodologies have proven to be useful (Martin et al. 2023). Moreover, depleting dominant retinal cell populations (as shown in this study) or enriching a specific cell type using cell surface markers before conducting transcriptome sequencing offer additional viable approaches for high resolution investigations (Peng et al. 2019). We also encountered technical hurdles that prevented us from validating other promising target genes using in situ hybridization since many aging‐related changes primarily affected relatively small cell populations in the retina (e.g., cones and horizontal cells).

## 4 Materials and Methods

### 4.1 Animals

C57BL/6J mouse strain (3‐, 8‐, 12‐, 18‐, and 24‐month‐old) were obtained from aged rodent colonies of the National Institute on Aging (NIA). This study conformed to the ARVO statement for the Use of Animals in Ophthalmic and Vision Research. Animal protocols were approved by the National Eye Institute (NEI) Animal Care and Use Committee (NEI‐ASP#650).

### 4.2 Single Cell Suspensions of Retina

Mouse retinas were dissected and dissociated as previously described (Fadl et al. 2020). Two aging datasets were created: the first using retinas from 3‐, 12‐, 18‐, and 24‐month‐old male mice to produce whole retina (WR) single‐cell suspensions (*N* = 4) or depleted rods (CD73N) (*N* = 4), and the second using cell suspensions from the superior (Sup) and inferior (Inf) retinal regions obtained from one‐millimeter punches of 3‐, 8‐, 12‐, and 24‐month‐old male retinas (*N* = 4). In the first set, whole retina (WR) single‐cell suspensions featured an abundance of rod photoreceptors, whereas anti‐CD73 conjugated magnetic beads were employed to deplete CD73‐positive cells (specifically rod photoreceptors), resulting in an enrichment of non‐rod cells in the retina samples (referred as CD73 negative, CD73N). CD73, a cell surface marker of mature rod cells (Koso et al. 2009), has been shown to be effective in depleting rods from retinal cell suspensions (Peng et al. 2019). Briefly, dissociated cells were incubated with anti‐mouse CD73‐APC conjugated antibodies (Milteny Biotec, clone REA778), followed by anti‐APC microbeads (Miltenyi Biotec, 130‐090‐855) to deplete rods. Incubations were performed at 4°C for 15 min. CD73‐negative cells were then selected using MS cell columns through an octoMACS Separator (Miltenyi Biotec).

### 4.3 Single‐Cell RNA Sequencing (scRNA‐Seq)

Single cell suspensions of the WR/CD73N retina samples were utilized as inputs for the 10x Genomics Chromium controller using v3.1 chemistry (Chromium Next GEM Single Cell 3′ GEM, Library and Gel Bead Kit v3.1). Approximately 17,000 live cells per sample were loaded into the Chromium chip following the Cell Suspension Volume Calculator Table to capture transcripts from approximately 10,000 cells. Libraries were performed according to the manufacturer's instructions (Single Cell 3′ v3.1 protocol, 10x Genomics) and sequenced on a Nova‐seq 6000 platform (Illumina, San Diego, CA, USA). Single cell suspensions of the Sup and Inf retinal region samples were utilized as inputs for the 10x Genomics Chromium controller using v2 chemistry (Chromium Next GEM Single Cell 3′ GEM, Library and Gel Bead Kit v2). Approximately 10,000 live cells per sample were loaded into the Chromium chip following the Cell Suspension Volume Calculator Table to capture transcripts from approximately 6000 cells. Libraries were performed according to the manufacturer's instructions (Single Cell 3′ v2 protocol, 10x Genomics) and sequenced on a Nova‐seq 6000 platform.

### 4.4 scRNA‐Seq Data Analysis

The WR/CD73N dataset and the Sup/Inf retina dataset were analyzed separately. Demultiplexing, barcoded processing, gene counting, and aggregation were made using the Cell Ranger software v7.0.1 and refdata‐gex‐mm10‐2020‐A as reference. Data matrixes were imported into Seurat v4.3.0 (Satija et al. 2015) in the R v4.2.1 statistical environment. For the WR/CD73N dataset, we performed initial matrix filtering of > 5 cells per gene, > 500 features per cell, and cells with mitochondria reads < 20%. For efficient ambient RNA removal, sample count matrixes of the CD73‐depleted and whole retina samples from the same age were merged using the ‘merge’ function in Seurat, and ambient RNA was removed using decontX function in the celda v1.14.2 package. DecontX adjusted count matrixes were split into individual samples prior to doublet detection and removal using DoubletFinder v2.0.3 with a target doublet rate of 7.5%. Since the dissociations from WR or CD73N at all timepoints were performed on the same days and all libraries were constructed and sequenced simultaneously, no batch effect was observed, or correction needed (data not shown).

Sample decontX adjusted count matrixes were merged into a single Seurat object, normalized, and variance stabilized using SCTransform v2 (Choudhary and Satija 2022). PCA (50 dimensions), FindNeighbors (30 dimensions), RunUMAP (30 dimensions), and FindClusters (original Louvain algorithm, res = 0.8) were performed using Seurat. The PCA and clustering settings were chosen to minimize cluster number while ensuring that the resulting clusters corresponded to sub cell types within cone photoreceptors and cone bipolar cells. Cell types were assigned using scType (https://github.com/IanevskiAleksandr/sc‐type) with sctype ‘eye’ database “Astrocytes”, “Immune cells”, “Retinal pigment epithelial cells”, and custom cell type gene lists (https://github.com/NEI‐NNRL/2023_Mouse_Retina_Aging/blob/main/src/Retina_Cell_type_MB_v2.xlsx). High resolution clustering (112 clusters) was performed using FindClusters(res = 6) to assess and remove putative doublet clusters exhibiting expression of canonical cell type markers from more than one cell type, however, undetected by DoubletFinder. Additionally, the rod photoreceptors were clustered with Monocle3 (Trapnell et al. 2014) using the Seurat normalized SCT count values as input. In the preprocess_cds function the following settings were used: num_dim = 100, norm_method = “none”, and pseudo_count = 0. Root cells were defined using 50 cells with the lowest UMAP_1 value from the Seurat UMAP. The defined root cells corresponded to the rods featuring the highest expression levels of phototransduction genes and *Nrl*. For the analysis of the Sup/Inf dataset, the initial matrix filtering criteria was reduced to > 200 features/cell due to the lower sequencing depth. The DecontX processing was performed on each sample prior to running DoubletFinder. Merging of decontX adjusted count matrix, normalization, and clustering were carried out as in case of the WR/CD73N dataset.

Previously reported mouse scRNA‐seq and single‐nucleus RNA sequencing (snRNA‐seq) data (GEO accession # GSE153674 and GSE132229 (Fadl et al. 2020; Heng et al. 2019)) were processed from fastq files using Cell Ranger and refdata‐gex‐mm10‐2020‐A as reference. Human scRNA‐seq (ArrayExpress accession number E‐MTAB‐7316 and GEO accession number GSE148077 (Lukowski et al. 2019; Yan et al. 2020)) and Macaque scRNA‐seq datasets (Genome Sequence Archive accession # CRA002680 and GEO accession # GSE118546 and GSE118852 (Yi et al. 2021; Peng et al. 2019)) were processed from fastq files using Cell Ranger with refdata‐gex‐GRCh38‐2020‐A and NCBI RefSeq Mmul_10 assembly GCF_003339765.1 as reference, respectively. Each data set was imported into separate Seurat objects and processed independently using an initial matrix filtering of > 5 cells per gene, > 200 features per cell, and cells with mitochondria reads < 20%, prior to running decontX. DecontX adjusted count matrixes were then merged into a single Seurat object, normalized, and variance stabilized using SCTransform v2. The merged data matrix was split by the individual of origin to generate the sample data and integration was performed as indicated in the Seurat v4.3 vignette “Performing integration on datasets normalized with SCTransform” (https://satijalab.org/seurat/archive/v4.3/integration_introduction). PCA (50 dimensions), FindNeighbors (30 dimensions), RunUMAP (30 dimensions), and FindClusters (res = 2) were executed prior to cell type determination using scType, with the previously mentioned custom cell type gene list.

### 4.5 Differential Gene Expression Analyses

Differential gene expression (DGE) was determined at the cell type and/or sub‐cell type level for the age comparisons tested using the Wilcoxon Rank Sum test in the Seurat function, FindMarkers. For each pairwise comparison, the data were subset for a particular cell type and ages investigated. DGE was performed using the log‐normalized decontX adjusted counts with the following settings in FindMarkers: min.pct = 0.25, logfc.threshold = log(1.5). Additionally, DGE gene lists were filtered for those having an adjusted *p*‐value < 1%.

### 4.6 Pathway Analysis

Functional gene enrichment of DGE results was performed using enrichR v3.1 with the “GO_Biological_Process_2021” database as pathway reference. The results were filtered for pathways having and adjusted *p*‐value < 5%. To reduce the redundancy of pathways inherent in GO analysis results, pathways were filtered for the most child term of any parent–child term passing significance.

### 4.7 scRNA Visualization

UMAP plots were rendered in Seurat using the PCA reduction of the SCT expression values. Dot plots were generated in Seurat using scaled average expression of SCT expression values. Violin plots were produced in Seurat using SCT expression values. Parallel plots of expression over aging for specific cell types were created using a custom script from scaled, average SCT expression values.

### 4.8 RNAscope In Situ Hybridization

RNAscope fluorescent in situ hybridization was accomplished on fresh‐frozen retina tissue from 3‐ and 24‐month‐old C57Bl/6J mice of both sexes. Briefly, the mice were sacrificed via CO_2_ inhalation, and eyes were rapidly enucleated, embedded in cryomolds with Tissue‐Tek O.C.T. Compound (Sakura Finetek, Torrance, CA, USA), and frozen in an isopentane bath cooled by dry ice. Fresh‐frozen eyes were stored at −80°C until further processing. The sections (12 μm thickness) were obtained on the superior–inferior or nasal‐temporal axes using a Thermo Scientific/Epredia CryoStar NX70 cryostat (Thermo Fisher Scientific, Waltham, MA, USA) and mounted on Superfrost Plus glass slides (Thermo Fisher Scientific). Three‐ and 24‐month‐old sections from the same area of the retina were mounted side‐by‐side. RNAscope hybridizations were carried out using RNAscope HiPlex12 reagent kit v2 (488, 550, 650) (Advanced Cell Diagnostics (ACD), Newark, CA, USA) following a slightly modified version of the manual.

RNAscope HiPlex probes (from ACD) were used to detect mRNA from the mouse genes *Bsg*, *Cacna2d1*, *Dlg2*, *Dpp10*, *Drd4*, *Gulp1*, *Immp2l*, *Rbm3*, and *Tafa3*. Fresh‐frozen retinal cryosections were fixed in prechilled 4% paraformaldehyde solution in 1X phosphate‐buffered saline solution (PBS) for 15 min at room temperature. The slides were washed twice in 1X PBS and dehydrated in sequential incubations with ethanol (50%, 70%, and 100%) for 5 min each at room temperature. After drying the slides for 5 min, retinal sections were treated with Protease IV for 30 min at room temperature and washed twice in 1X PBS. Appropriate combinations of hybridization probes were incubated for 2 h at 40°C, followed by three amplification steps, 4,6‐diamidino‐2‐phenylindole (DAPI) counterstaining, and mounting with Fluoromount‐G mounting medium (Thermo Fisher Scientific). The slides were then imaged using a Zeiss LSM 880 confocal microscope (Zeiss, Germany) with identical settings across young and old sections and represented as maximum intensity projections of acquired confocal z‐stacks. Analysis was performed with the Advanced Cell Diagnostics' Image Registration Software and ImageJ.

## Author Contributions

L.C., conceptualization, formal analysis, validation, investigation, methodology, writing – original draft, writing – review and editing; M.J.B., conceptualization, formal analysis, computational analysis, writing – original draft, writing – review and editing; B.R.F., investigation, methodology, writing – review and editing; H.S.C., investigation, methodology, writing – review and editing; S.P., computational analysis, writing – review and editing; A.S., conceptualization, resources, supervision, funding acquisition, writing – original draft, project administration, writing – review and editing.

## Conflicts of Interest

The authors declare no conflicts of interest.

## Supporting information


Figure S1.

Figure S2.

Figure S3.

Figure S4.

Figure S5.

Figure S6.

Figure S7.

Figure S8.

Figure S9.

Figure S10.

Table S1.

Table S2.

Table S3.

Table S4.

Table S5.

Table S6.

Table S7.

Table S8.

Table S9.

Table S10.

Table S11.

Table S12.

Table S13.

Table S14.

## Data Availability

The raw and final analyzed data will be available in NCBI's Gene Expression Omnibus (GEO) data repository with accession numbers: GSE230049 and GSE256389. Custom code used for data analysis is available in GitHub (https://github.com/NEI‐NNRL/2023_Mouse_Retina_Aging).
