# Supplementary figures and images for: Transcriptional Heterogeneity and Differential Response of Rod Photoreceptor Pathway Uncovered by Single‐Cell RNA Sequencing of the Aging Mouse Retina
Source: Aging Cell. 2025 Feb 15;24(5):e70001. doi: 10.1111/acel.70001 (PMC12073905; doi:10.1111/acel.70001)

Figure S1

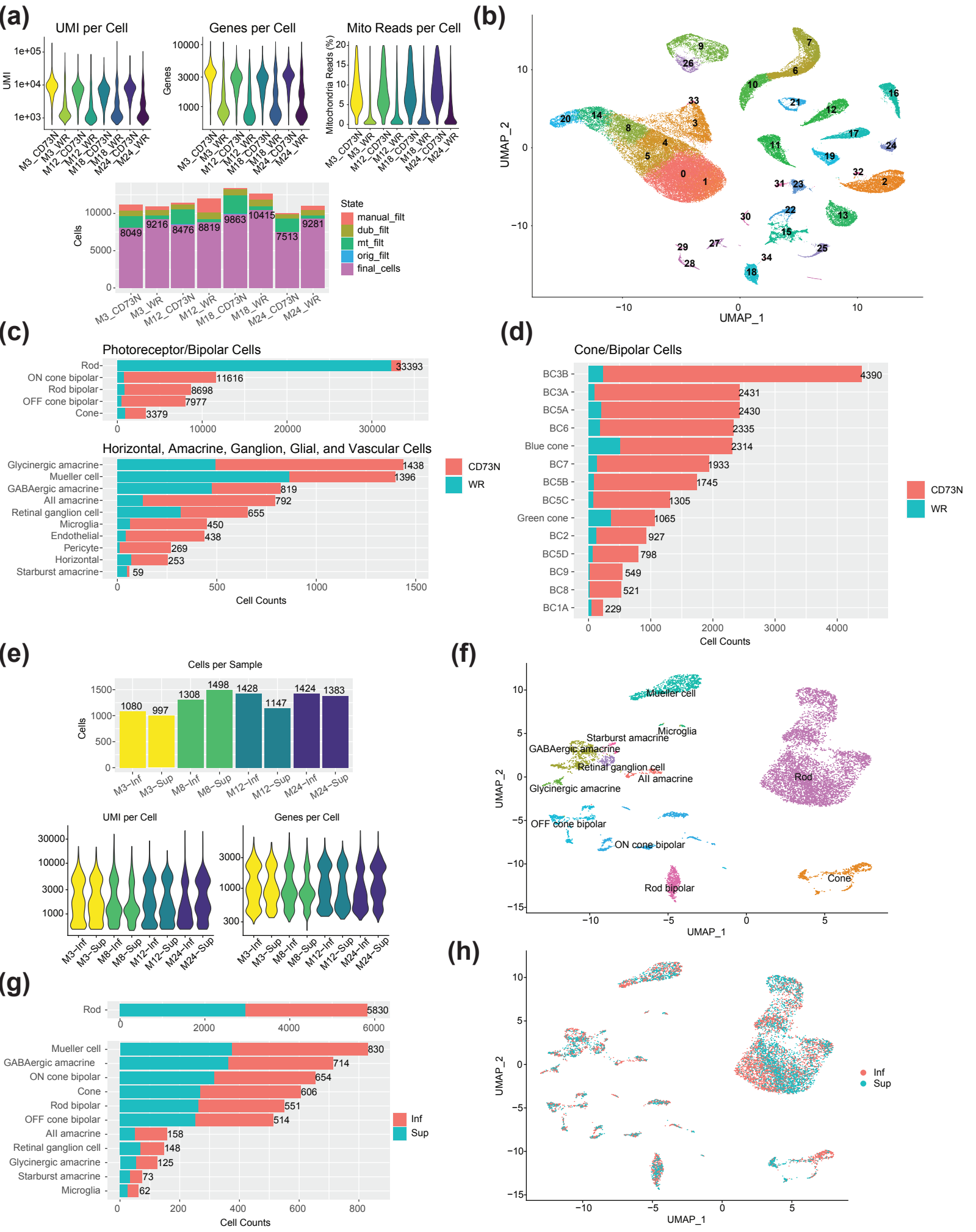

Supplement: Supplementary file 1 — Figure S1. [file ACEL-24-e70001-s015.pdf]

**(a)**

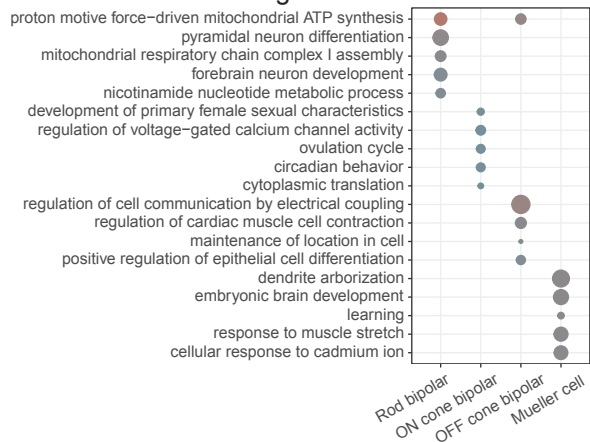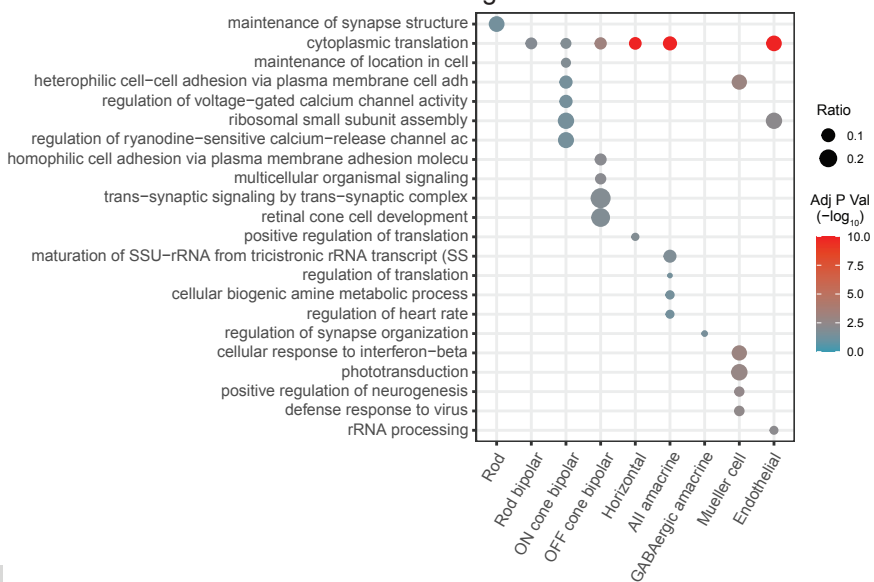

**(b)**

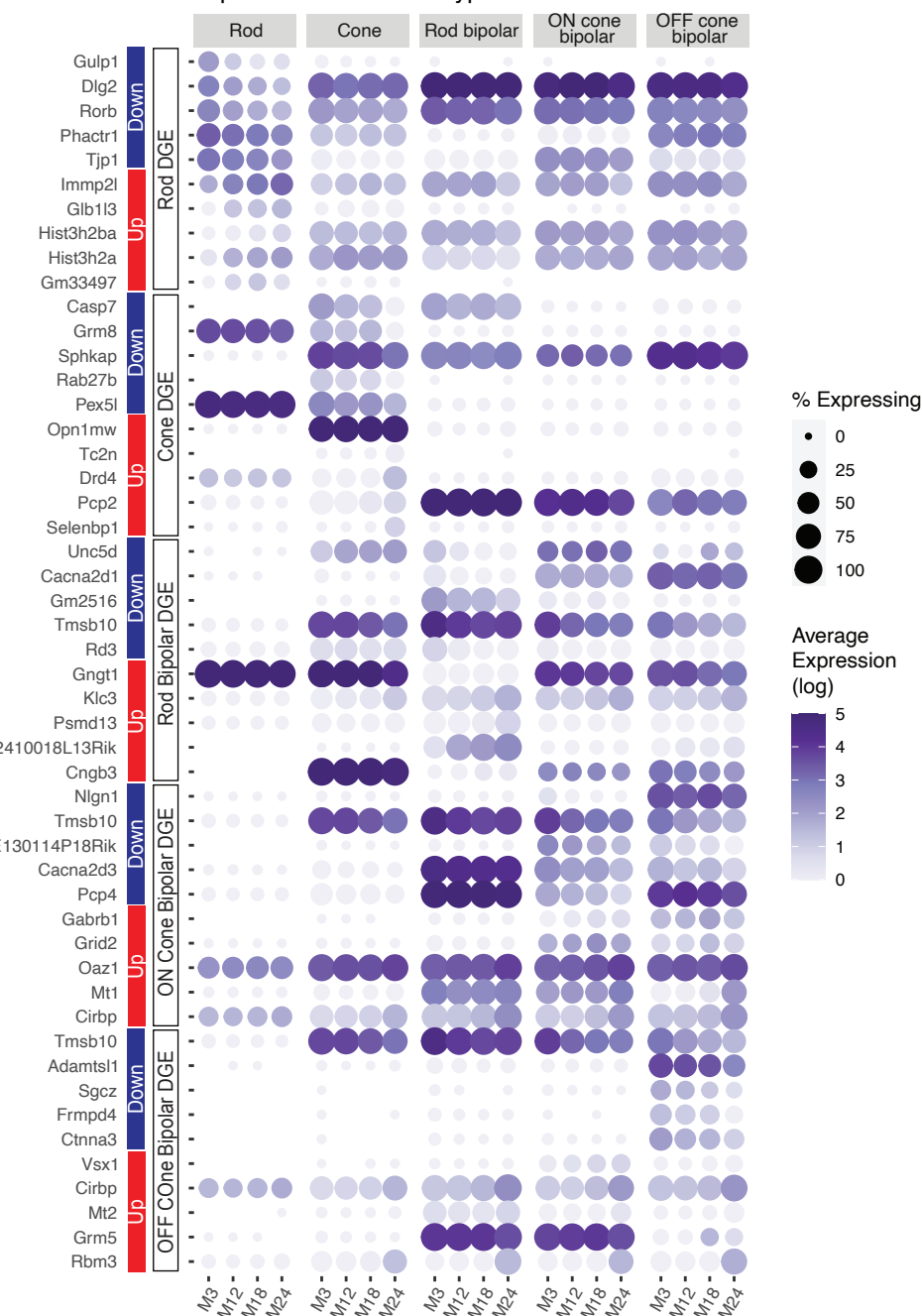

Supplement: Supplementary file 2 — Figure S2. [file ACEL-24-e70001-s020.pdf]

Figure S3

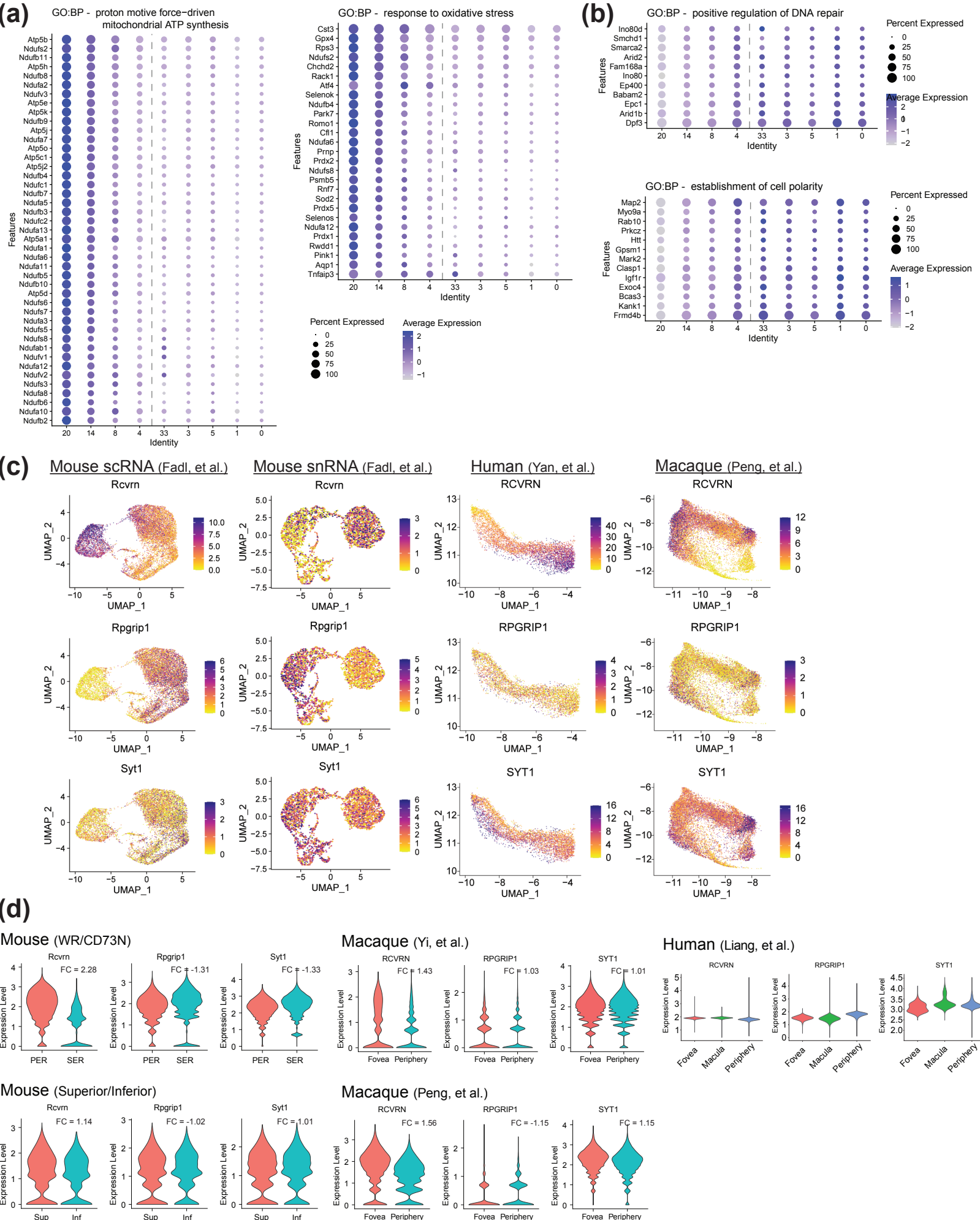

Supplement: Supplementary file 3 — Figure S3. [file ACEL-24-e70001-s006.pdf]

# Figure S4

## WR/CD73N

(a) (b)

### PER Markers

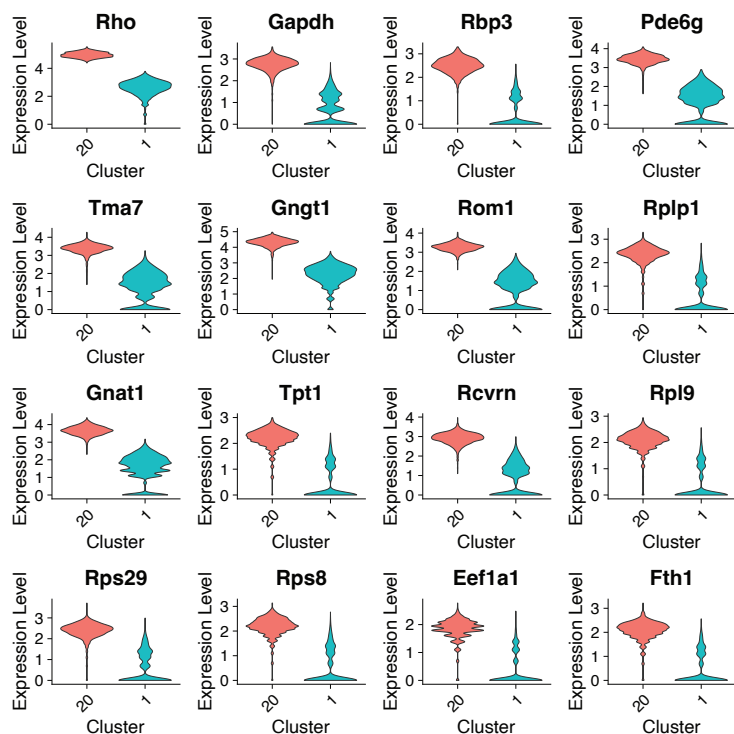

### SER Markers

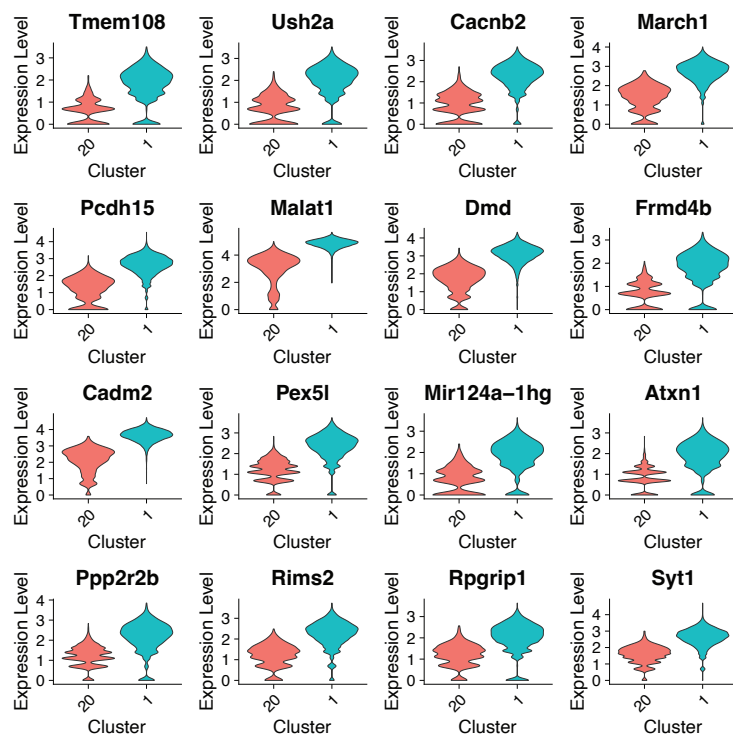

## Sup/Inf Retina

(c) (d)

### PER Markers

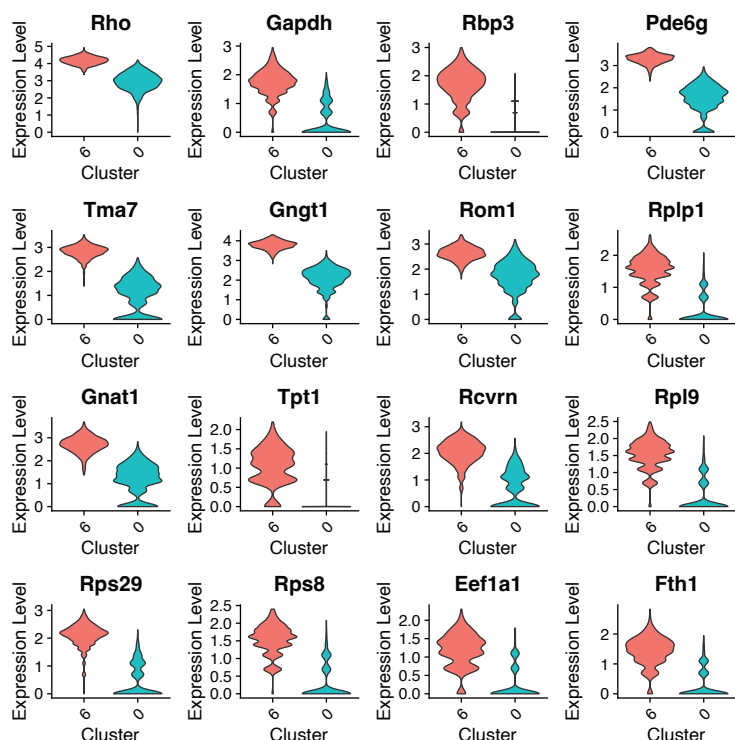

### SER Markers

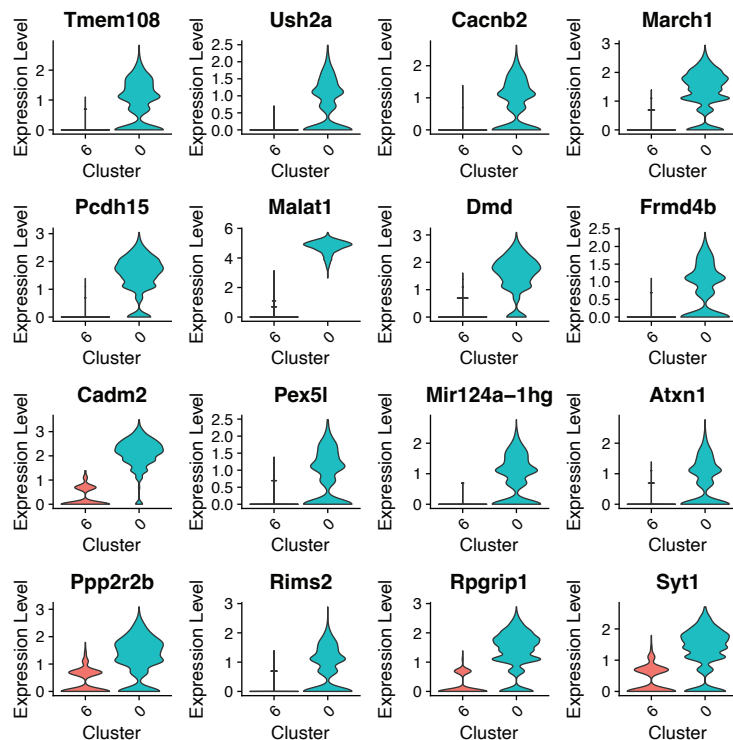

Supplement: Supplementary file 4 — Figure S4. [file ACEL-24-e70001-s010.pdf]

Figure S5

Mouse Superior/Inferior

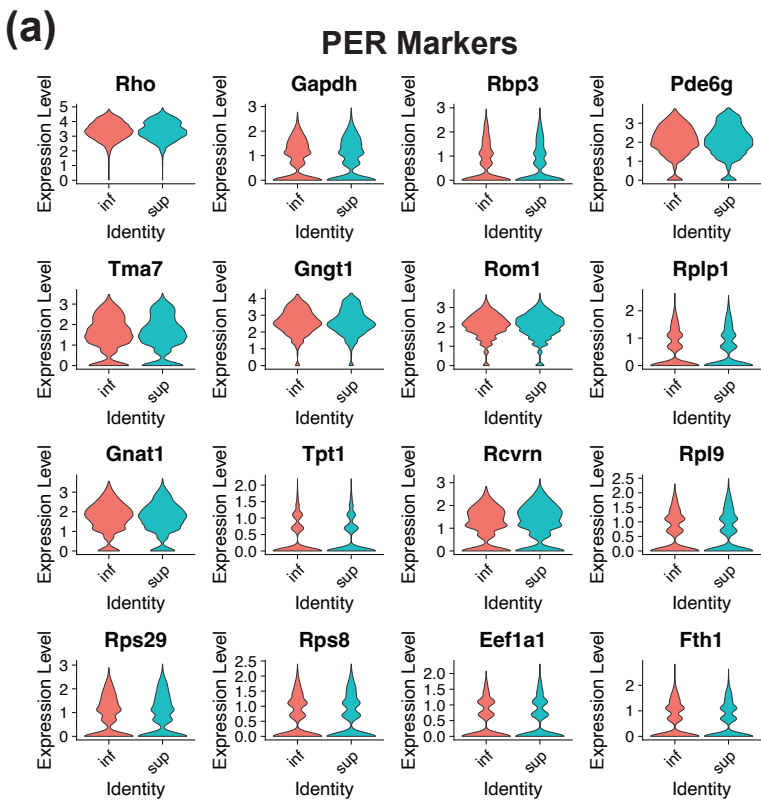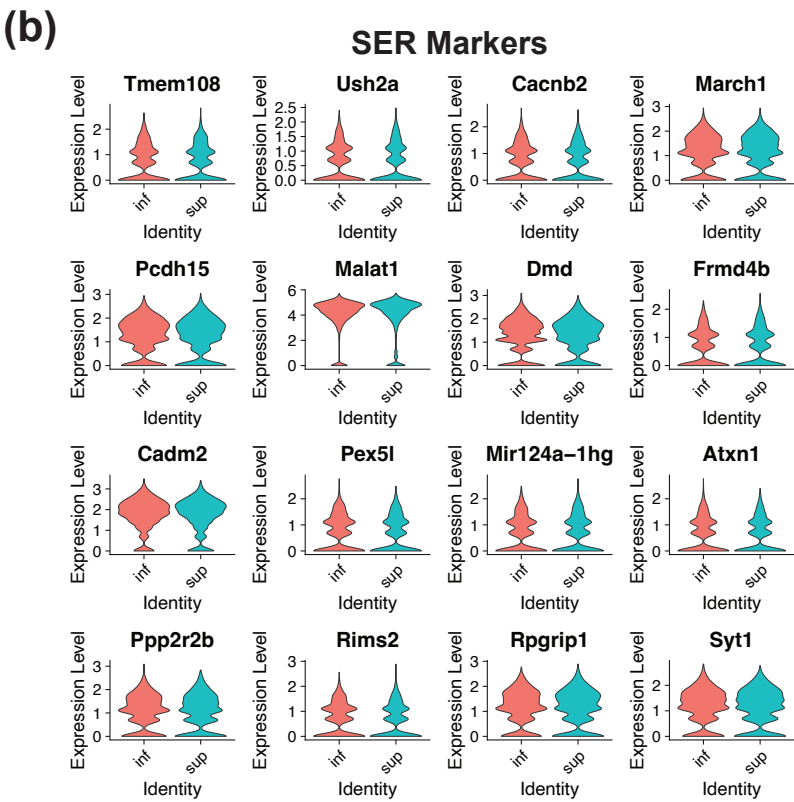

Human (Liang, et al.)

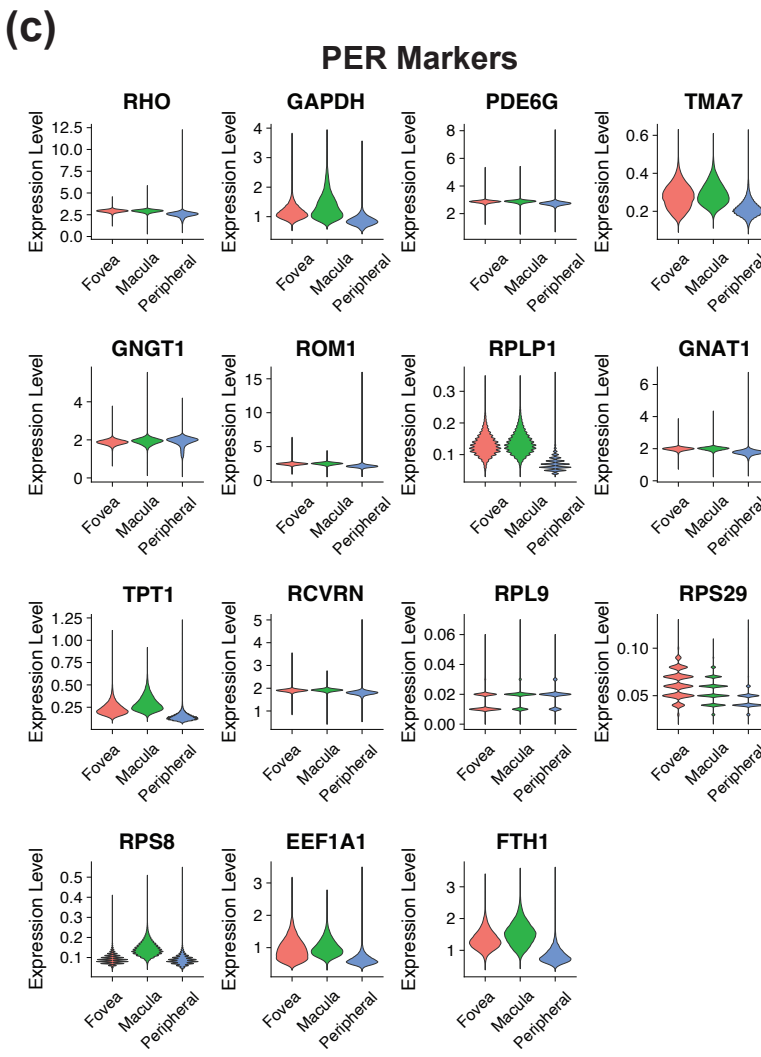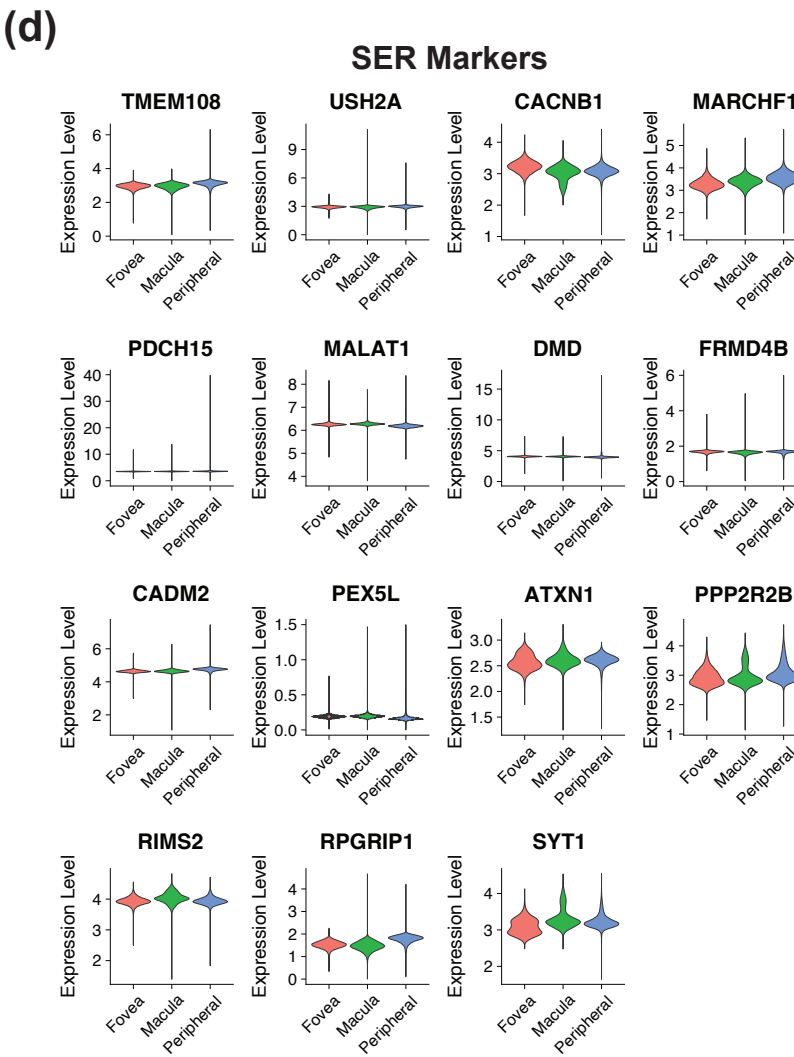

Supplement: Supplementary file 5 — Figure S5. [file ACEL-24-e70001-s002.pdf]

# Figure S6

## Macaque (Yi, et al.)

(a)

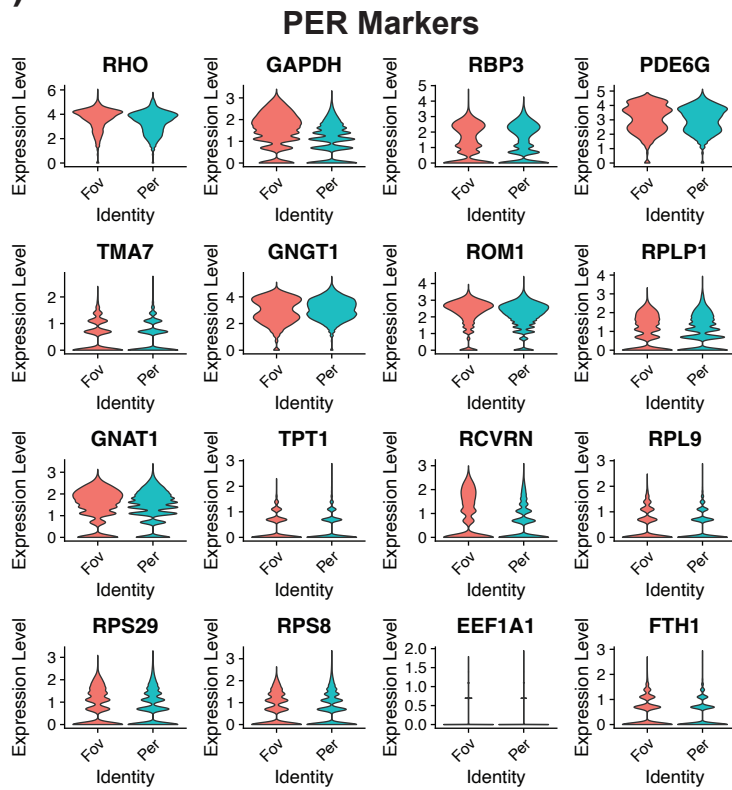

(b)

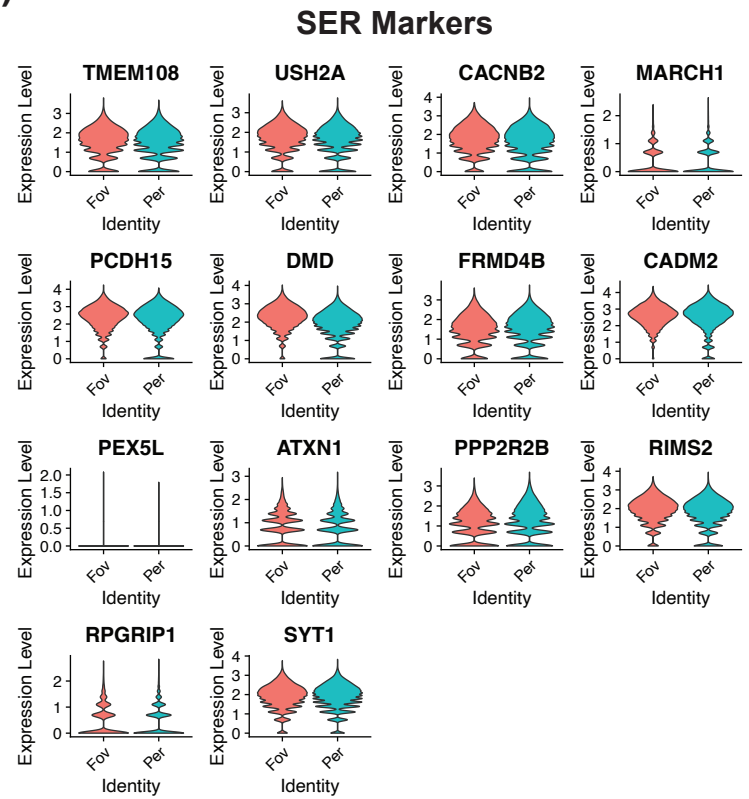

## Macaque (Peng, et al.)

(c)

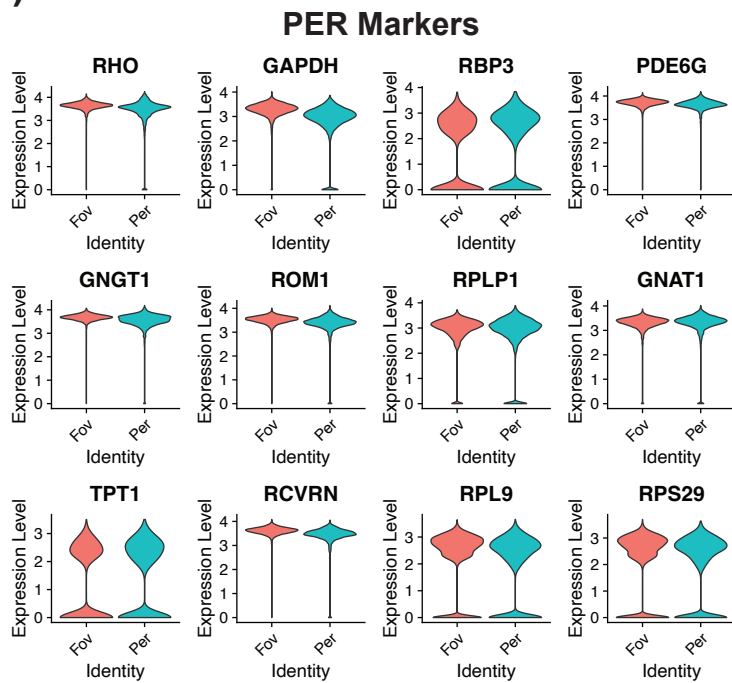

(d)

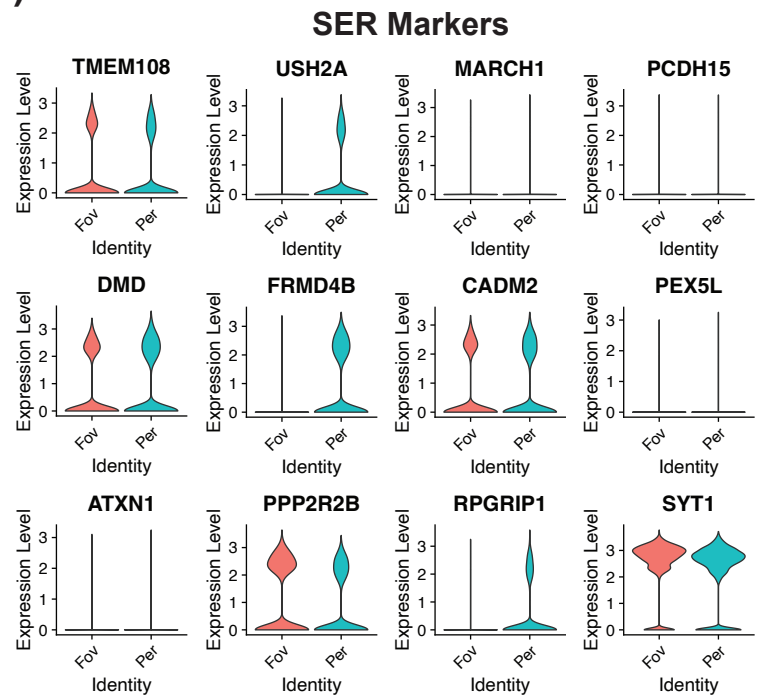

Supplement: Supplementary file 6 — Figure S6. [file ACEL-24-e70001-s023.pdf]

Figure S7

(a)

PER

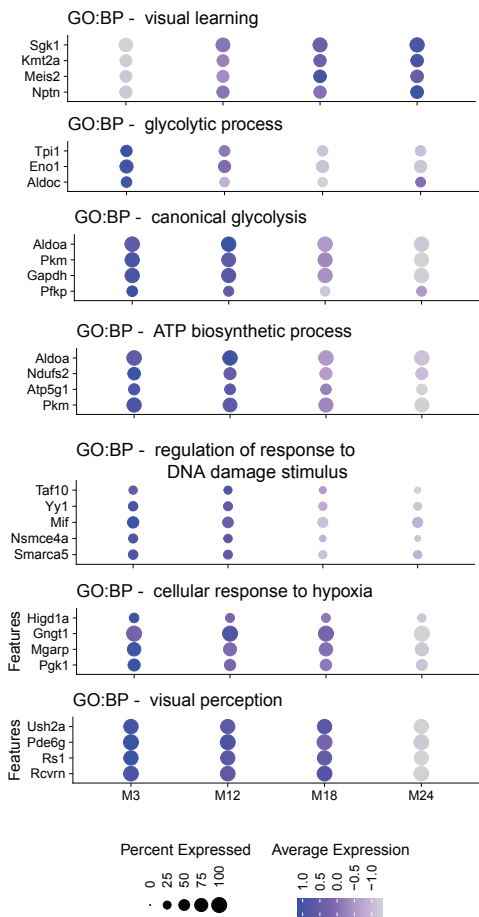

(b)

SER

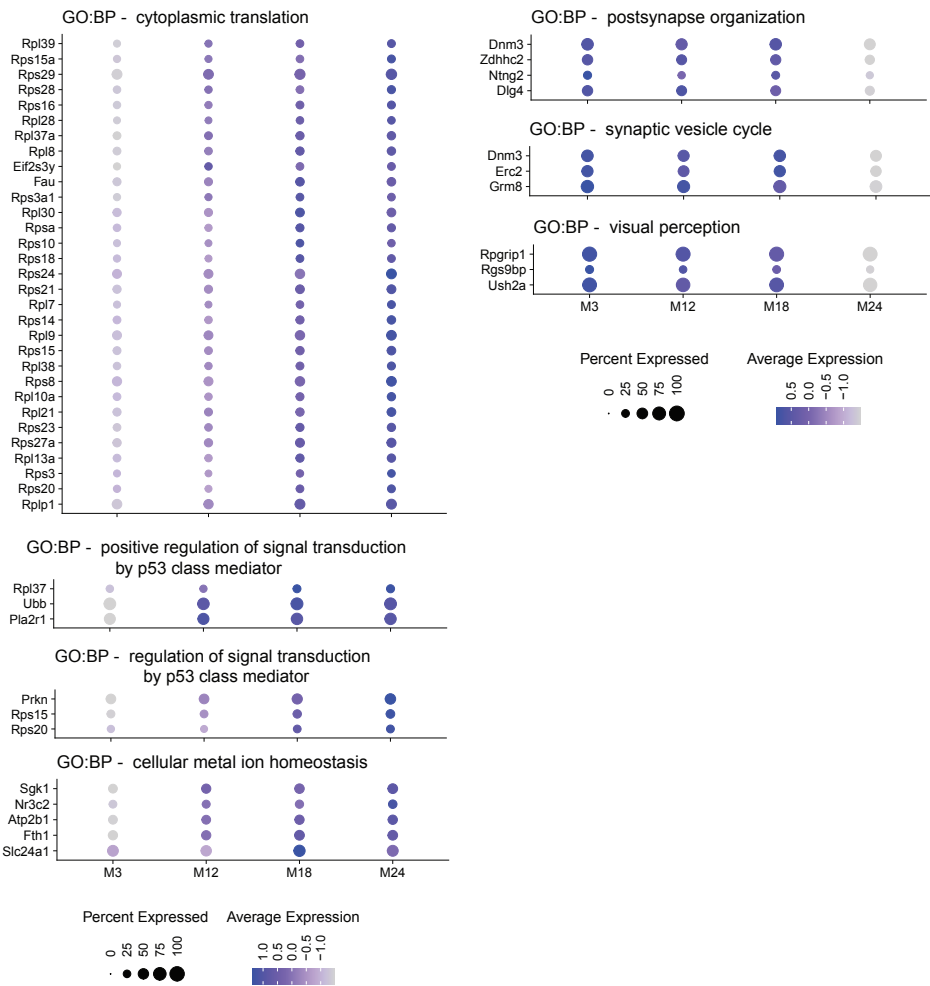

(c)

Dpp10

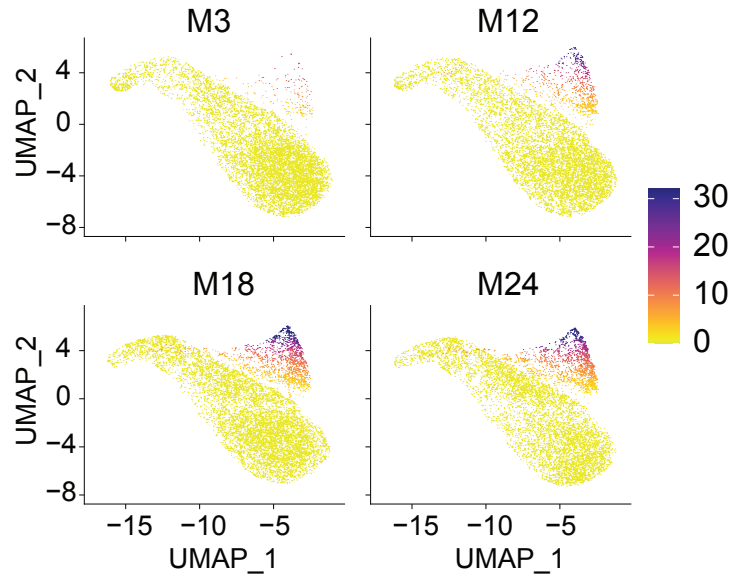

Supplement: Supplementary file 7 — Figure S7. [file ACEL-24-e70001-s013.pdf]

Figure S8

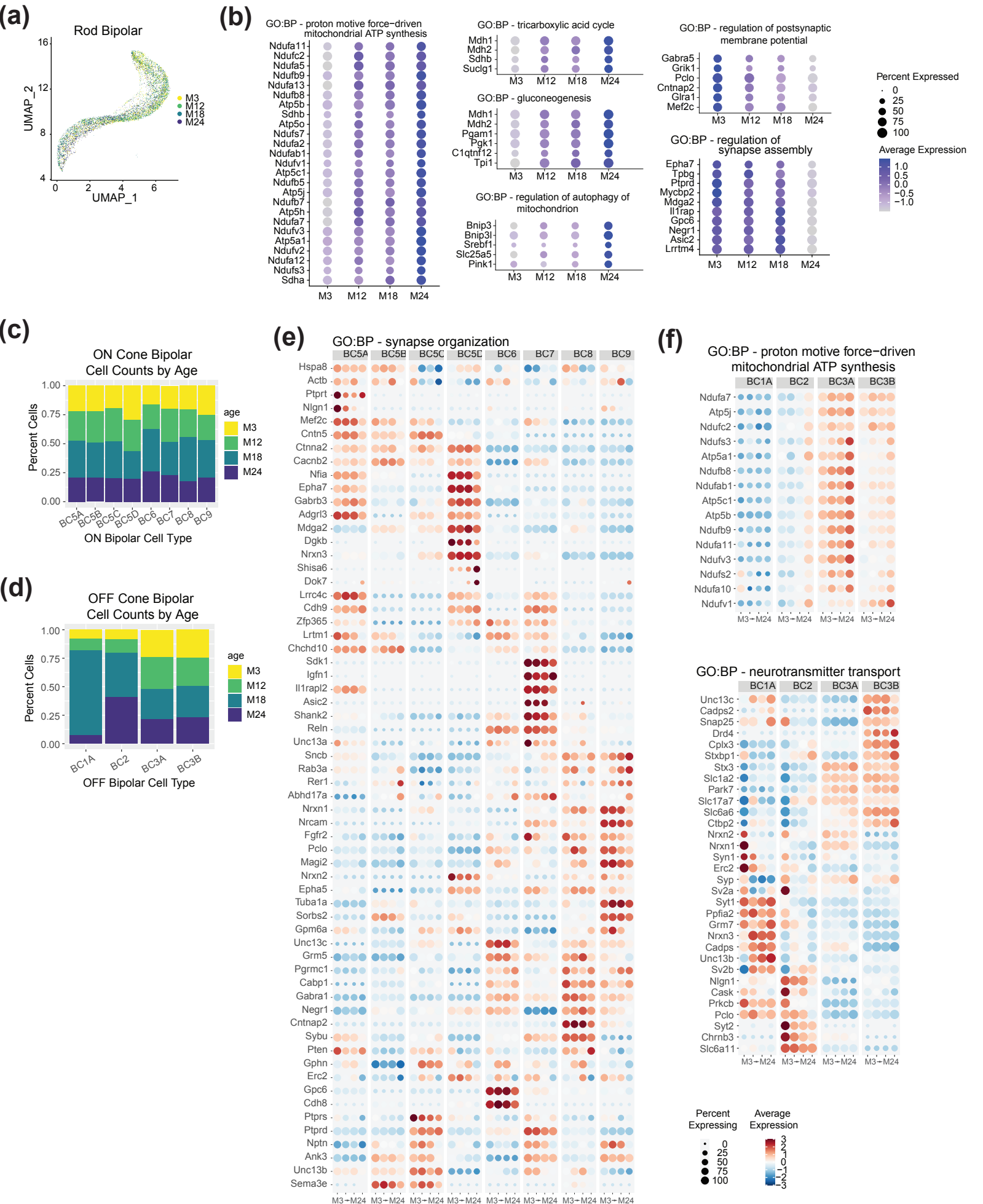

Supplement: Supplementary file 8 — Figure S8. [file ACEL-24-e70001-s008.pdf]

Figure S9

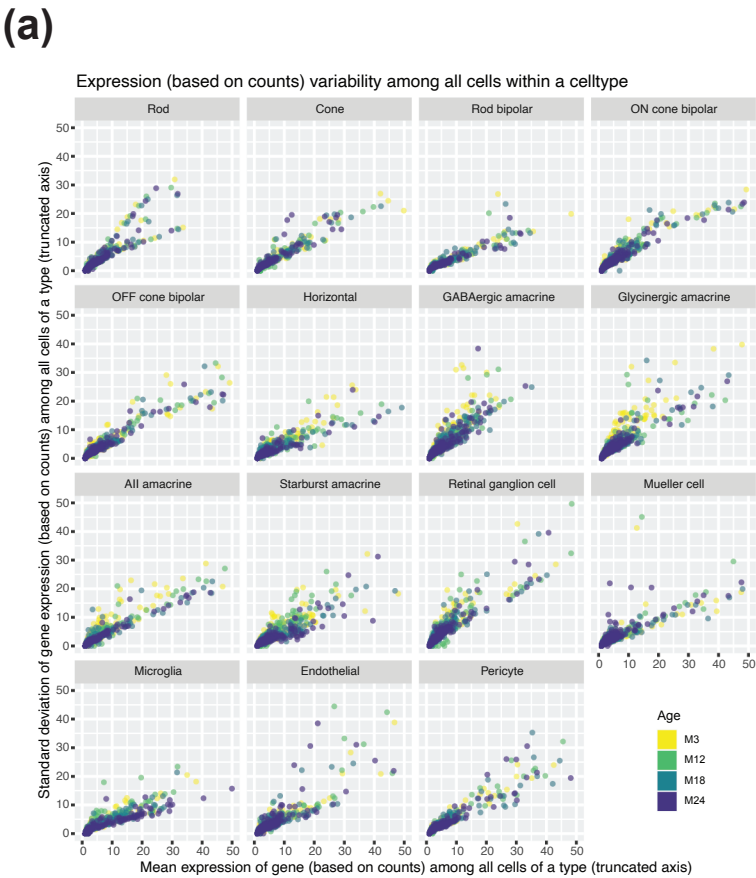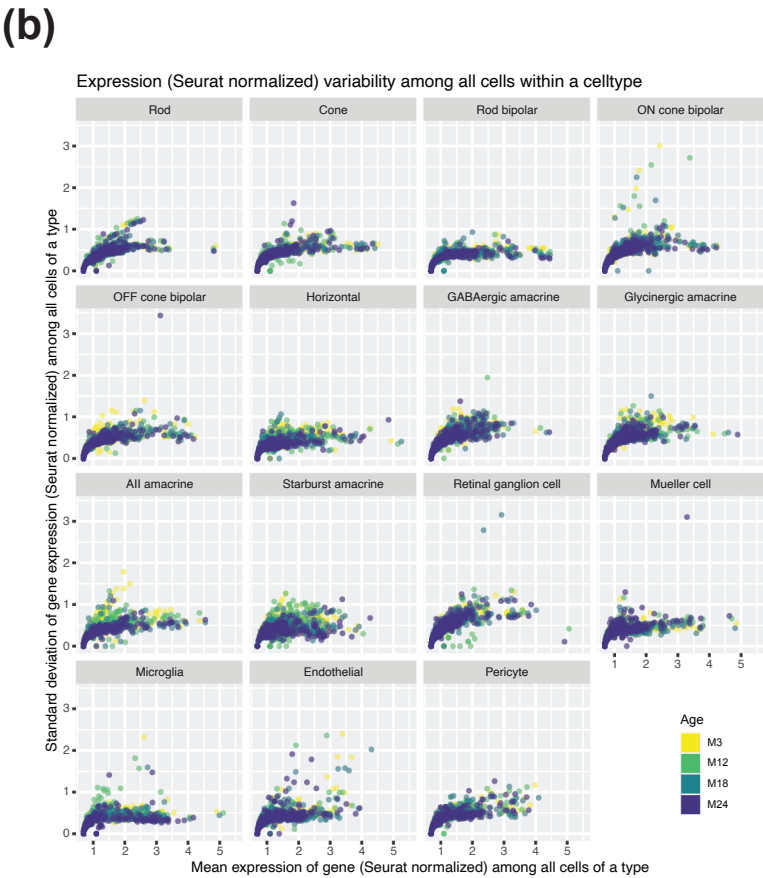

Supplement: Supplementary file 9 — Figure S9. [file ACEL-24-e70001-s014.pdf]

Figure S10

(a)

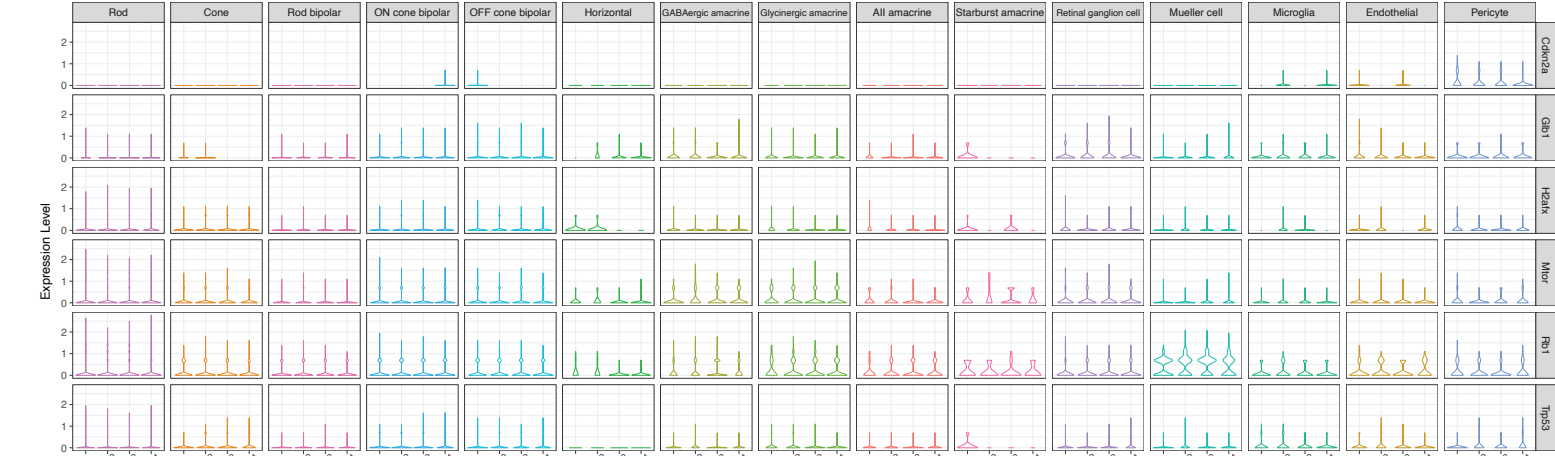

(b)

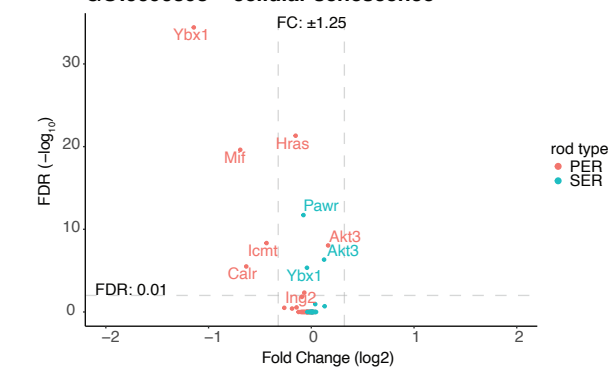

(c)

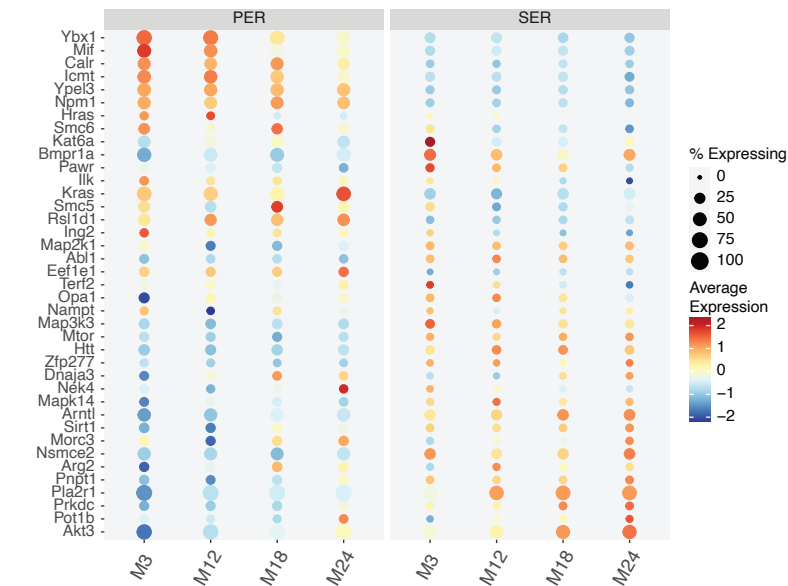

(d)

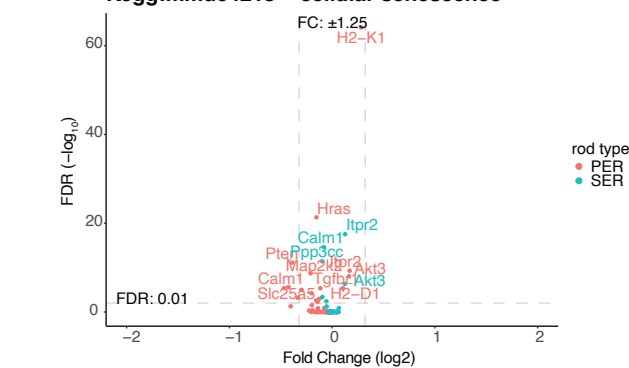

(e)

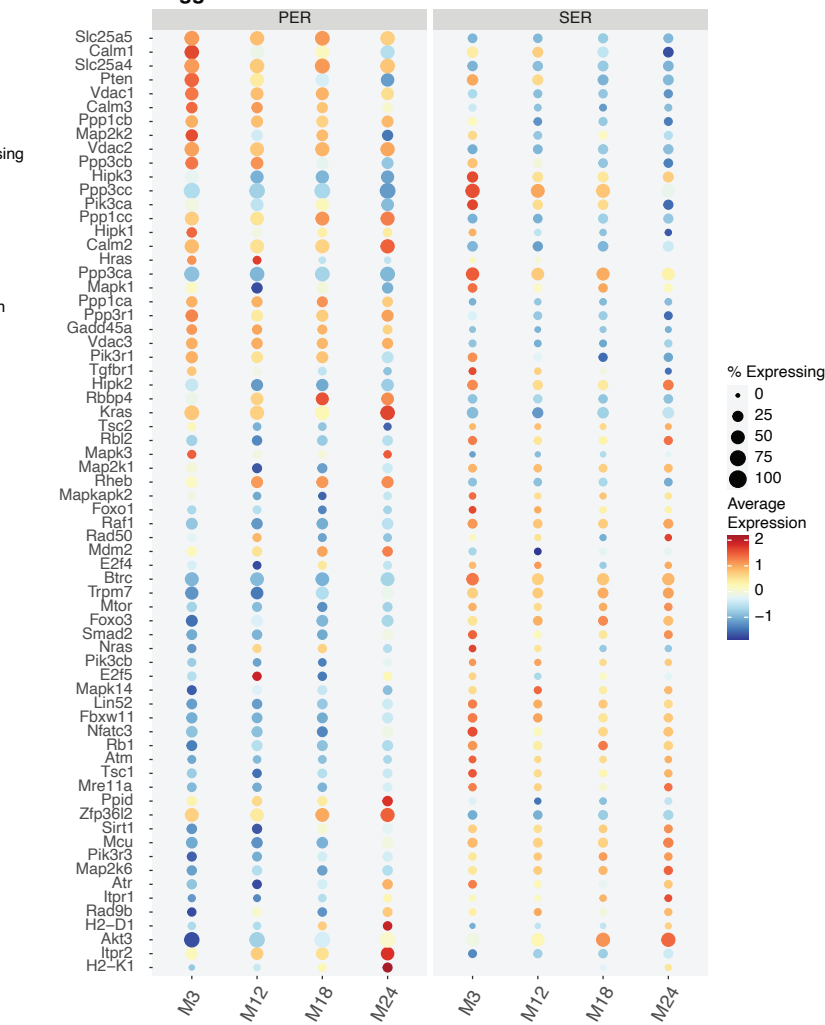

Supplement: Supplementary file 10 — Figure S10. [file ACEL-24-e70001-s022.pdf]
